# Risk of diabetic ketoacidosis of SGLT2 inhibitors in patients with type 2 diabetes: a systematic review and network meta-analysis of randomized controlled trials

**DOI:** 10.3389/fphar.2023.1145587

**Published:** 2023-06-13

**Authors:** Shiwen Yang, Ying Liu, Shengzhao Zhang, Fengbo Wu, Dan Liu, Qingfang Wu, Hanrui Zheng, Ping Fan, Na Su

**Affiliations:** ^1^ Department of Pharmacy, West China Hospital of Sichuan University, Chengdu, China; ^2^ Department of Pharmacy, Karamay Central Hospital, Karamay, China; ^3^ Department of Endocrinology and Metabolism, West China Hospital of Sichuan University, Chengdu, China; ^4^ West China School of Pharmacy, Sichuan University, Chengdu, China

**Keywords:** sodium–glucose cotransporter 2 inhibitors, type 2 diabetes mellitus, diabetic ketoacidosis, network meta-analysis, placebo

## Abstract

**Background:** Sodium–glucose cotransporter-2 (SGLT2) inhibitors have proven to be effective in improving glycemic control in patients with type 2 diabetes mellitus (T2DM). However, the risk of diabetic ketoacidosis (DKA) in patients remains unclear. The purpose of this study is to conduct this systematic review and network meta-analysis for the risk of DKA of SGLT2 inhibitors in patients with T2DM.

**Methods:** We searched for randomized controlled trials (RCTs) concerning SGLT2 inhibitors in patients with T2DM in PubMed, EMBASE (Ovid SP), Cochrane Central Register of Controlled Trials (Ovid SP), and ClinicalTrials.gov from inception to January 2022. The primary outcomes were the risk of DKA. We assessed the sparse network with a fixed-effect model and consistency model in a frequentist framework with a graph-theoretical method by the netmeta package in R. We assessed the evidence quality of evidence of outcomes according to the Grading of Recommendations Assessment, Development, and Evaluation (GRADE).

**Results:** In total, 36 studies involving 52,264 patients were included. The network showed that there was no significant difference observed among SGLT2 inhibitors, other active antidiabetic drugs, and placebo in the risk of DKA. There was no significant difference in the DKA risk between different doses of SGLT2 inhibitors. The certainty of the evidence ranged from very low to moderate. The probabilities of rankings and P-score showed that compared to placebo, SGLT2 inhibitors might increase the risk of DKA (P-score = 0.5298). Canagliflozin might have a higher DKA risk than other SGLT2 inhibitors (P-score = 0.7388).

**Conclusion:** Neither SGLT2 inhibitors nor other active antidiabetic drugs were associated with an increased risk of DKA compared to placebo, and the risk of DKA with SGLT2 inhibitors was not found to be dose-dependent. In addition, the use of canagliflozin was less advisable than other SGLT2 inhibitors according to the rankings and P-score.

**Systematic Review Registration:** https://www.crd.york.ac.uk/prospero/, identifier PROSPERO, CRD42021297081.

## 1 Introduction

Type 2 diabetes mellitus (T2DM), the most common type of chronic disease characterized by hyperglycemic metabolism, has become a public health problem, prevalence and incidence of which have been increasing in recent years (Katsiki et al., 2020; Zhou et al., 2021). T2DM may cause irreversible damage to the heart and blood vessels, kidneys, and eyes (Chiang et al., 2021). At present, sodium-glucose cotransporter 2 (SGLT2) inhibitors are novel therapeutic targets for the treatment of T2DM through inhibiting glucose reabsorption in renal proximal convoluted tubules, reducing blood glucose fluctuations, promoting urinary glucose excretion, improving insulin sensitivity and *ß*-cell function in the liver and peripheral tissues, and further improving hepatic insulin resistance (Li et al., 2020; Scheen, 2020). In addition, SGLT2 inhibitors can exert the protective effect of the cardiovascular system and kidney, and delay the occurrence and development of T2DM complications by affecting blood lipid, weight loss, and blood pressure reduction (Häring et al., 2013; Li et al., 2021; Zheng et al., 2021; Zou et al., 2022).

Diabetic ketoacidosis (DKA) rarely occurs spontaneously in people with T2DM, but when appeared, it might be associated with the use of certain drugs (American Diabetes Association Professional Practice Committee, 2022). In May 2015, the Food and Drug Administration (FDA) issued a drug safety bulletin warning that canagliflozin, dapagliflozin, and empagliflozin could lead to hospitalization in patients with T2DM due to DKA (FDA, 2015a). However, according to a joint statement issued by the American Society of Clinical Endocrinologists (AACE) and the Endocrine Society (ACE), patients with T2DM treated with SGLT2 inhibitors have no higher risk of DKA than the general population, and there was no clear evidence that SGLT2 inhibitors are associated with DKA in T2DM (Handelsman et al., 2016). It remains unclear whether SGLT2 inhibitors increase the risk of DKA compared with other active antidiabetic drugs until now, and the risk of DKA among different doses of SGLT2 inhibitors also remains unknown. Therefore, we conducted this systematic review and network meta-analysis of the available evidence for the risk of DKA of SGLT2 inhibitors in patients with T2DM.

## 2 Methods

We conducted this systematic review and network meta-analysis in accordance with the Preferred Reporting Items for Systematic Reviews and Meta-analyses (PRISMA). This network meta-analysis was registered on the International Prospective Register of Systematic Review.

### 2.1 Literature search and eligible criteria

We comprehensively searched PubMed, EMBASE (Ovid SP), and Cochrane Central Register of Controlled Trials (Ovid SP) for studies published from the time when the databases were established to 26 January 2022. ClinicalTrial.gov was screened for unpublished studies. The reference lists of relevant published research studies investigating the risk of DKA of SGLT2 inhibitors in patients with T2DM were also screened for potentially relevant studies. The key terms searched in this study were based on the PICOS framework (Supplementary Table S1 and Supplementary Table S2). Duplicate records were removed with EndNote X9.

### 2.2 Study selection

We included studies meeting the following criteria: 1) participants: adults (>18 years) with a diagnosis of T2DM; 2) interventions/comparisons: SGLT2 inhibitors, active antidiabetic drugs (we defined active antidiabetic drugs as antidiabetic drugs other than SGLT2 inhibitors), or placebo; 3) outcomes: reporting the risk of DKA; and 4) study design: published or unpublished randomized controlled trials (RCTs) limited to the English language. The exclusion criteria were as follows: 1) including pregnant participants; 2) animal experiments; 3) studies published in a language other than English; 4) published as abstract only; 5) including patients with prediabetes; and 6) DKA caused by T2DM.

### 2.3 Screening process and data extraction

All retrieved literature studies were identified by two independent reviewers (YL and SY), and data were extracted by a predefined form. Any discrepancies were resolved by discussion with a third reviewer (NS), as required. We extracted the data including the first author’s name, publication year, sample size, follow-up length, intervention and comparison, outcomes, and the characteristics of participants.

SGLT2 inhibitors with diverse doses were separated to several trials. If a study contained more than one SGLT-2 inhibitor or more than one dose of SGLT-2 inhibitors, we defined them as a different comparison.

### 2.4 Quality assessment and the certainty of evidence

Four reviewers (N.S., P.F., Y.L., and S.Y.) conceived the study. Two independent reviewers (Y.L. and S.Y.) assessed the risk of bias of all included studies according to ROB 2, a revised Cochrane risk-of-bias tool for randomized trials (Sterne et al., 2019), and the discrepancies were resolved by consulting the third reviewer (N.S.). The Grading of Recommendations, Assessment, Development, and Evaluation (GRADE) was used to assess the certainty of the evidence for the outcome. Three reviewers (F.W., D.L., and S.Z.) conducted the analysis and interpreted the data. Two reviewers (Q.W. and H.Z.) checked the analysis data on the review. The members of the research team assessed the confidence rating for each comparison as high, moderate, low, or very low, based on the direct and indirect estimates. Discrepancies were resolved by discussions.

### 2.5 Treatment nodes

Treatment nodes were grouped by different kinds of active antidiabetic drugs and different doses of SGLT2 inhibitors. We drew network plots with the *multinma* package in R (version 4.1.3) (Multinma, 2020).

### 2.6 Statistical analysis

We conducted a network meta-analysis of randomized controlled trials that assessed the sparse network with a fixed-effect model (Efthimiou et al., 2019) and consistency model in the frequentist framework with a graph-theoretical method by the netmeta package in R (version 4.1.3) (Rücker et al., 2015). The effect size for assessing DKA safety was calculated as odds ratios (ORs) with accompanying 95% credible intervals (CIs). We calculated the consistency by node-splitting models (Van Valkenhoef et al., 2016). We calculated the P-score to rank treatments (Salanti et al., 2011). We assessed the global and local statistical heterogeneity with generalized Cochran’s Q. We estimated the variance in heterogeneity between studies using the DerSimonian–Laird random-effects model. We assessed transitivity using descriptive statistics from studies and population baselines (Cipriani et al., 2013).

We assessed publication bias using funnel plots and Egger’s test with the netmeta package in R. Multiple sensitivity analyses were carried out to assess the robustness of the final results, including the following: 1) this analysis was estimated in a Bayesian framework; 2) exclusion of studies with treatment duration <24 weeks; 3) exclusion of studies without a placebo control; 4) exclusion of the high risk of bias studies (exclusion of unblinded studies.); 5) exclusion of studies where the risk of DKA was 0 percent; and 6) exclusion of studies with fewer than 100 participants.

## 3 Results

### 3.1 Characteristics of eligible studies

**FIGURE 1:**
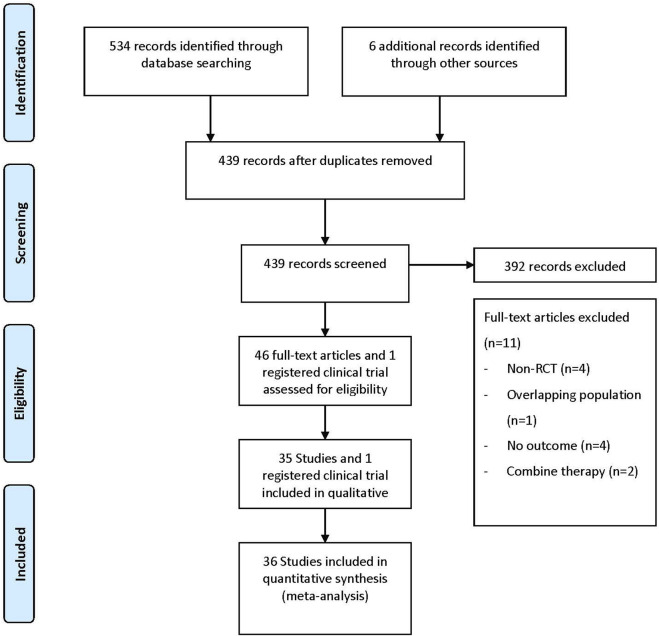
Flow diagram for study identification and inclusion.

The literature search flow diagram is shown in Figure 1. After screening 534 articles and 6 registered clinical trials, a total of 36 studies between 2013 and 2021 were included in the meta-analysis according to predetermined criteria (35 articles and 1 registered clinical trial), involving 52,264 patients and 70 DKA events (Bode et al., 2013; Lavalle-González et al., 2013; NCT, 2013; Barnett et al., 2014; Rosenstock et al., 2014; Erondu et al., 2015; Haering et al., 2015; Roden et al., 2015; Rosenstock et al., 2015; Araki et al., 2016; Frías et al., 2016; Hadjadj et al., 2016; Mancia et al., 2016; Rodbard et al., 2016; Rosenstock et al., 2016; Ito et al., 2017; Neal et al., 2017; Søfteland et al., 2017; Aronson et al., 2018; Dagogo-Jack et al., 2018; Fioretto et al., 2018; Han et al., 2018; Hollander et al., 2018; Kawamori et al., 2018; Pratley et al., 2018; Ridderstrale et al., 2018; Rosenstock et al., 2018; Scott et al., 2018; Terauchi et al., 2018; Allegretti et al., 2019; Cho et al., 2019; Ji et al., 2019; Perkovic et al., 2019; Pollock et al., 2019; Cahn et al., 2020; Persson et al., 2021; Weng et al., 2021). Of the included studies, 21 were conducted in multinational country studies, and most studies were registered (35/36, 97%) and all published in English. The baseline characteristics of the included studies are presented in Table 1 and Supplementary Table S3, where 34 were two-arm studies and two were three-arm studies. Among the 36 studies (the retrieved SGLT2 inhibitors contain 1 study about bexagliflozin; 7 studies about canagliflozin; 8 studies about dapagliflozin; 10 studies about empagliflozin; 6 studies about ertugliflozin; 1 study about henagliflozin; 2 studies about ipragliflozin; and 1 study about tofogliflozin), 19 studies with different doses of SGLT2 inhibitors were compared; 27 studies compared SGLT2 inhibitors to placebo; 11 studies compared SGLT2 inhibitors to active antidiabetic drugs (active antidiabetic drugs retrieved include pioglitazone, exenatide, glimepiride, metformin, and sitagliptin); and two studies compared active drugs to placebo. The study population comprised 31,829 males (60.9%) and 20,435 females (39.1%), with the mean age being 59.3 years (ranging from 51.6 to 69.9 years); the mean HbA1c was 8.1% (ranging from 6.9% to 9.3%); the baseline mean BMI was 30.4 kg/m^2^ (ranging from 25.4 to 35.0 kg/m^2^); the mean disease duration was 9.8 years (ranging from 3.3 to 17.7 years); and the mean duration of treatment was 61.7 weeks (ranging from 12.0 to 271.0 weeks). In addition, most trials were funded by pharmaceutical companies (34/36, 94%).

**TABLE 1:** Characteristic baseline of randomized controlled trials.

First author	Register number/trial name	Location	No. of patients(n)	N	Intervention	Age (years) (mean ± SD)	HbA1c (%) (mean ± SD)	BMI (kg/m^2^) (mean ± SD)	Duration of diabetes (years) (mean ± SD)	Length of the follow-up (weeks)
Allegretti et al. (2019)	NCT02836873	Multinational	312	157	Bexagliflozin 20 mg/day	69.30 ± 8.36	8.01 ± 0.79	30.29 ± 5.99	15.54 ± 9.20	24
				155	Placebo	69.90 ± 8.29	7.95 ± 0.81	30.10 ± 5.77	16.28 ± 8.98	
Araki et al. (2016)	NCT02157298	Japan	183	123	Dapagliflozin 5 mg/day	58.30 ± 9.80	8.30 ± 0.80	26.90 ± 4.90	15.30 ± 9.00	16
				60	Placebo	57.60 ± 9.90	8.50 ± 0.90	26.10 ± 3.50	14.20 ± 8.90	
Aronson et al. (2018)	NCT01958671	Multinational	461	156	Ertugliflozin 5 mg/day	56.80 ± 11.40	8.16 ± 0.88	33.20 ± 7.40	5.11 ± 5.09	52
				152	Ertugliflozin 15 mg/day	56.20 ± 10.80	8.35 ± 1.12	32.50 ± 5.70	5.22 ± 5.55	
				153	Placebo	56.10 ± 10.90	8.11 ± 0.92	33.30 ± 6.80	4.63 ± 4.52	
Barnett et al. (2014)	NCT01164501	Multinational	741	98	Empagliflozin 10 mg/day	63.20 ± 8.50	8.02 ± 0.84	-	-	52
				322	Empagliflozin 25 mg/day	63.90 ± 9.00	7.96 ± 0.73			
				321	Placebo	64.10 ± 8.70	8.09 ± 0.80			
Bode et al. (2013)	NCT01106651	Multinational	714	241	Canagliflozin 100 mg/day	64.30 ± 6.50	7.80 ± 0.80	31.40 ± 4.40	12.30 ± 7.80	104
				236	Canagliflozin 300 mg/day	63.40 ± 6.00	7.70 ± 0.80	31.50 ± 4.60	11.30 ± 7.20	
				237	Placebo	63.20 ± 6.20	7.80 ± 0.80	31.80 ± 4.80	11.40 ± 7.30	
Cahn et al. (2020)	NCT01730534	United States of America	17,160	8,582	Dapagliflozin 10 mg/day	63.90 ± 6.80	8.30 ± 1.20	32.10 ± 6.00	11.00	271
				8,578	Placebo	64.00 ± 6.80	8.30 ± 1.20	32.10 ± 6.10	10.00	
Cho et al. (2019)	UMIN000022804	Japan	71	36	Dapagliflozin 5 mg/day	63.10 ± 10.00	6.90 ± 0.60	28.70 ± 6.20	-	24
				35	Pioglitazone 10–30 mg/day	63.60 ± 10.20	6.90 ± 0.60	28.50 ± 4.20		
Dagogo-Jack et al. (2018)	NCT02036515	United States of America	462	156	Ertugliflozin 5 mg/day	59.20 ± 9.30	8.10 ± 0.90	31.20 ± 5.50	9.90 ± 6.10	52
				153	Ertugliflozin 15 mg/day	59.70 ± 8.60	8.00 ± 0.80	30.90 ± 6.10	9.20 ± 5.30	
				153	Placebo	58.30 ± 9.20	8.00 ± 0.90	30.30 ± 6.40	9.40 ± 5.60	
Fioretto et al. (2018)	NCT2413398	Multinational	321	160	Dapagliflozin 10 mg/day	65.30 ± 6.22	8.33 ± 1.08	32.60 ± 4.70	14.30 ± 8.10	24
				161	Placebo	66.20 ± 6.49	8.03 ± 1.08	31.60 ± 5.00	14.50 ± 8.30	
Frías et al. (2016)	NCT02229396	Multinational	457	227	Exenatide 2 mg/day	54.00 ± 10.00	9.30 ± 1.10	32.00 ± 5.90	7.40 ± 5.50	28
				230	Dapagliflozin 10 mg/day	55.00 ± 9.09	9.30 ± 1.00	33.00 ± 6.10	7.10 ± 5.50	
Hadjadj et al. (2016)	NCT01719003	Multinational	665	164	Empagliflozin 25 mg/day	53.30 ± 10.70	8.86 ± 1.29	30.60 ± 5.90	-	26
				169	Empagliflozin 10 mg/day	53.10 ± 10.70	8.62 ± 1.24	30.30 ± 5.20		
				164	Metformin 2000 mg/day	51.60 ± 10.80	8.58 ± 1.13	30.50 ± 5.90		
				168	Metformin 1000 mg/day	53.40 ± 10.90	8.69 ± 1.04	30.30 ± 5.80		
Haering et al. (2015)	NCT01289990	Multinational	666	225	Empagliflozin 10 mg/day	57.00 ± 9.20	8.20 ± 0.80	28.30 ± 5.40	-	76
				216	Empagliflozin 25 mg/day	57.40 ± 9.30	8.10 ± 0.80	28.30 ± 5.50		
				225	Placebo	56.90 ± 9.20	8.10 ± 0.80	27.90 ± 4.90		
Han et al. (2018)	NCT02452632	Korean	139	73	Ipragliflozin 50 mg/day	57.62 ± 8.26	7.90 ± 0.69	25.50 ± 3.07	11.62 ± 5.89	24
				66	Placebo	57.44 ± 7.88	7.92 ± 0.79	26.05 ± 3.79	11.33 ± 6.63	
Hollander et al. (2018)	NCT01999218	Multinational	1,325	448	Ertugliflozin 5 mg/day	58.80 ± 9.70	7.80 ± 0.60	31.70 ± 5.50	7.40 ± 5.70	52
				440	Ertugliflozin 15 mg/day	58.00 ± 9.90	7.80 ± 0.60	31.30 ± 6.20	7.50 ± 5.70	
				437	Glimepiride 1-6/8 mg/day	57.80 ± 9.20	7.80 ± 0.60	31.20 ± 6.40	7.50 ± 5.60	
Ito et al. (2017)	UMIN000022651	Japan	66	32	Ipragliflozin 50 mg/day	57.30 ± 12.10	8.50 ± 1.50	29.90 ± 6.20	8.70 ± 5.80	24
				34	Pioglitazone 15–30 mg/day	59.10 ± 9.80	8.30 ± 1.40	30.70 ± 5.00	9.50 ± 5.80	
Ji et al. (2019)	NCT02630706	China	506	170	Ertugliflozin 5 mg/day	56.100 ± 9.00	8.10 ± 0.90	26.00 ± 2.80	7.00 ± 5.00	26
				169	Ertugliflozin 15 mg/day	56.30 ± 9.30	8.10 ± 0.90	25.70 ± 3.20	7.50 ± 5.10	
				167	Placebo	56.90 ± 9.00	8.10 ± 1.00	26.10 ± 3.40	6.40 ± 5.10	
Kawamori et al. (2018)	NCT02453555	Japan	275	182	Empagliflozin 10 mg/day	60.00 ± 9.90	8.36 ± 0.74	26.00 ± 3.80	9.00 ± 7.20	52
				93	Placebo	59.80 ± 10.80	8.27 ± 0.65	26.60 ± 4.50	8.70 ± 6.10	
Lavalle-González et al. (2013)	NCT01106677	Multinational	1,284	183	Placebo	55.30 ± 9.80	8.00 ± 0.90	31.10 ± 6.10	6.80 ± 5.30	52
				366	Sitagliptin 100 mg/day	55.50 ± 9.60	7.90 ± 0.90	32.00 ± 6.10	6.80 ± 5.20	
				368	Canagliflozin 100 mg/day	55.50 ± 9.40	7.90 ± 0.90	32.40 ± 6.40	6.70 ± 5.40	
				367	Canagliflozin 300 mg/day	55.30 ± 9.20	7.90 ± 0.90	31.40 ± 6.30	7.10 ± 5.40	
Mancia et al. (2016)	NCT01370005	United States of America	824	276	Empagliflozin 10 mg/day	60.60 ± 8.50	7.87 ± 0.77	32.40 ± 5.30	-	12
				276	Empagliflozin 25 mg/day	59.90 ± 9.70	7.92 ± 0.72	33.00 ± 5.00		
				272	Placebo	60.30 ± 8.80	7.90 ± 0.72	32.40 ± 4.90		
NCT (2013)	NCT01106625	Multinational	469	157	Canagliflozin 100 mg/day	57.30 ± 10.47	-	-	-	52
				156	Canagliflozin 300 mg/day	56.00 ± 8.95				
				156	Placebo	56.70 ± 8.36				
Neal et al. (2017)	NCT01032629 NCT01989754	Multinational	10,142	5,795	Canagliflozin 300 mg/day	63.20 ± 8.30	8.20 ± 0.90	31.90 ± 5.90	13.50 ± 7.70	188.2
				4,347	placebo	63.40 ± 8.20	8.20 ± 0.90	32.00 ± 6.00	13.70 ± 7.80	
Perkovic et al. (2019)	NCT02065791	Multinational	4,397	2,200	Canagliflozin 100 mg/day	62.90 ± 9.20	8.30 ± 1.30	31.40 ± 6.20	15.50 ± 8.70	130
				2,197	Placebo	63.20 ± 9.20	8.30 ± 1.30	31.30 ± 6.20	16.00 ± 8.60	
Persson et al. (2021)	NCT03036150	Multinational	2,906	1,455	Dapagliflozin 10 mg/day	64.10 ± 9.80	7.80 ± 1.70	30.20 ± 6.20	13.70 ± 7.10	164
				1,451	Placebo	64.70 ± 9.50	7.80 ± 1.60	30.40 ± 6.30	13.80 ± 7.50	
Pollock et al. (2019)	NCT02547935	Multinational	293	145	Dapagliflozin 10 mg/day	64.70 ± 8.60	8.44 ± 1.00	30.19 ± 5.30	17.55 ± 7.70	24
				148	Placebo	64.70 ± 8.50	8.57 ± 1.20	30.34 ± 5.60	17.71 ± 9.50	
Pratley et al. (2018)	NCT02099110	Multinational	745	250	Ertugliflozin 5 mg/day	55.10 ± 10.10	8.60 ± 1.00	31.80 ± 6.20	7.10 ± 5.40	52
				248	Ertugliflozin 15 mg/day	55.30 ± 9.50	8.60 ± 1.00	31.50 ± 5.80	7.30 ± 5.40	
				247	Sitagliptin 100 mg/day	54.80 ± 10.70	8.50 ± 1.00	31.70 ± 6.50	6.20 ± 5.20	
Ridderstråle et al. (2018)	NCT01167881	Multinational	1,545	765	Empagliflozin 25 mg/day	56.20 ± 10.30	7.92 ± 0.81	29.95 ± 5.28	-	208
				780	Glimepiride 1–4 mg/day	55.70 ± 10.40	7.92 ± 0.86	30.27 ± 5.30		
Rodbard et al. (2016)	-	Multinational	213	107	Canagliflozin 100 mg/day	57.40 ± 9.30	8.50 ± 0.90	32.30 ± 5.80	9.80 ± 5.40	26
				106	Placebo	57.50 ± 10.10	8.40 ± 0.80	31.70 ± 5.50	10.10 ± 5.90	
Roden et al. (2015)	NCT01289990 NCT01177813	Multinational	899	224	Empagliflozin 10 mg/day	56.20 ± 11.60	7.87 ± 0.88	28.30 ± 5.50	-	76
				224	Empagliflozin 25 mg/day	53.80 ± 11.60	7.86 ± 0.85	28.20 ± 5.50		
				223	Sitagliptin 100 mg/day	55.10 ± 9.90	7.85 ± 0.79	28.20 ± 5.20		
				228	Placebo	56.20 ± 10.90	7.91 ± 0.78	28.70 ± 6.20		
Rosenstock et al. (2016)	NCT01809327	Multinational	712	237	Canagliflozin 100 mg/day	54.00 ± 10.70	8.80 ± 1.20	32.40 ± 5.40	3.50 ± 4.40	26
				238	Canagliflozin 300 mg/day	55.80 ± 9.60	8.80 ± 1.20	32.60 ± 5.80	3.30 ± 4.40	
				237	Metformin	55.20 ± 9.80	8.80 ± 1.20	33.00 ± 6.00	3.30 ± 4.50	
Rosenstock et al. (2018)	NCT02033889	Multinational	621	207	Ertugliflozin 5 mg/day	56.60 ± 8.10	8.10 ± 0.90	30.80 ± 4.80	7.90 ± 6.10	26
				205	Ertugliflozin 15 mg/day	56.90 ± 9.40	8.10 ± 0.90	31.10 ± 4.50	8.10 ± 5.50	
				209	Placebo	56.50 ± 8.70	8.20 ± 0.90	30.70 ± 4.70	8.00 ± 6.30	
Rosenstock et al. (2014)	NCT01306214	United States of America	563	186	Empagliflozin 10 mg/day	56.70 ± 8.70	7.19 ± 0.08	34.70 ± 3.80	-	52
				189	Empagliflozin 25 mg/day	58.00 ± 9.40	7.09 ± 0.08	35.00 ± 4.00		
				188	Placebo	55.30 ± 10.10	7.48 ± 0.09	34.70 ± 4.30		
Rosenstock et al. (2015)	NCT01011868	Multinational	494	169	Empagliflozin 10 mg/day	58.60 ± 9.80	8.30 ± 0.80	32.10 ± 5.80	-	78
				155	Empagliflozin 25 mg/day	59.90 ± 10.50	8.30 ± 0.80	32.70 ± 5.90		
				170	Placebo	58.10 ± 9.40	8.20 ± 0.80	31.80 ± 6.00		
Scott et al. (2018)	NCT02532855	Multinational	613	307	Sitagliptin 100 mg/day	67.70 ± 8.50	7.70 ± 0.70	31.80 ± 5.70	10.50 ± 7.00	24
				306	Dapagliflozin 10 mg/day	66.60 ± 8.60	7.80 ± 0.70	31.50 ± 5.30	10.70 ± 7.40	
Søfteland et al. (2017)	NCT01734785	Multinational	327	109	Empagliflozin 10 mg/day	54.30 ± 9.60	7.97 ± 0.84	31.20 ± 5.90	-	24
				110	Empagliflozin 25 mg/day	55.40 ± 9.90	7.97 ± 0.82	29.90 ± 5.30		
				108	Placebo	55.90 ± 9.70	7.97 ± 0.85	29.60 ± 5.70		
Terauchi et al. (2018)	NCT02201004	Multinational	210	140	Tofogliflozin 20 mg/day	59.10 ± 10.90	8.53 ± 0.76	25.79 ± 3.46	15.06 ± 9.39	56
				70	placebo	56.4 ± 10.00	8.40 ± 0.65	26.89 ± 3.88	12.39 ± 7.34	
Weng et al. (2021)	NCT03159052	China	483	162	Henagliflozin 5 mg/day	54.30 ± 9.50	8.50 ± 0.80	25.50 ± 2.90	5.53 ± 4.96	24
				160	Henagliflozin 10 mg/day	54.70 ± 10.70	8.40 ± 0.90	25.60 ± 3.20	6.39 ± 4.80	
				161	Placebo	55.03 ± 9.50	8.50 ± 0.90	25.40 ± 3.10	6.58 ± 5.81	

**Footnotes:** HbA1c: hemoglobin A1c; BMI: body mass index.

### 3.2 Risk of bias of included studies

The overall risk of bias was low. The assessment of the risk of bias in the included studies is shown in Supplementary Table S4. Overall quality assessment indicated that more than half of the studies had a low risk of bias.

### 3.3 Results of network meta-analysis

**FIGURE 2:**
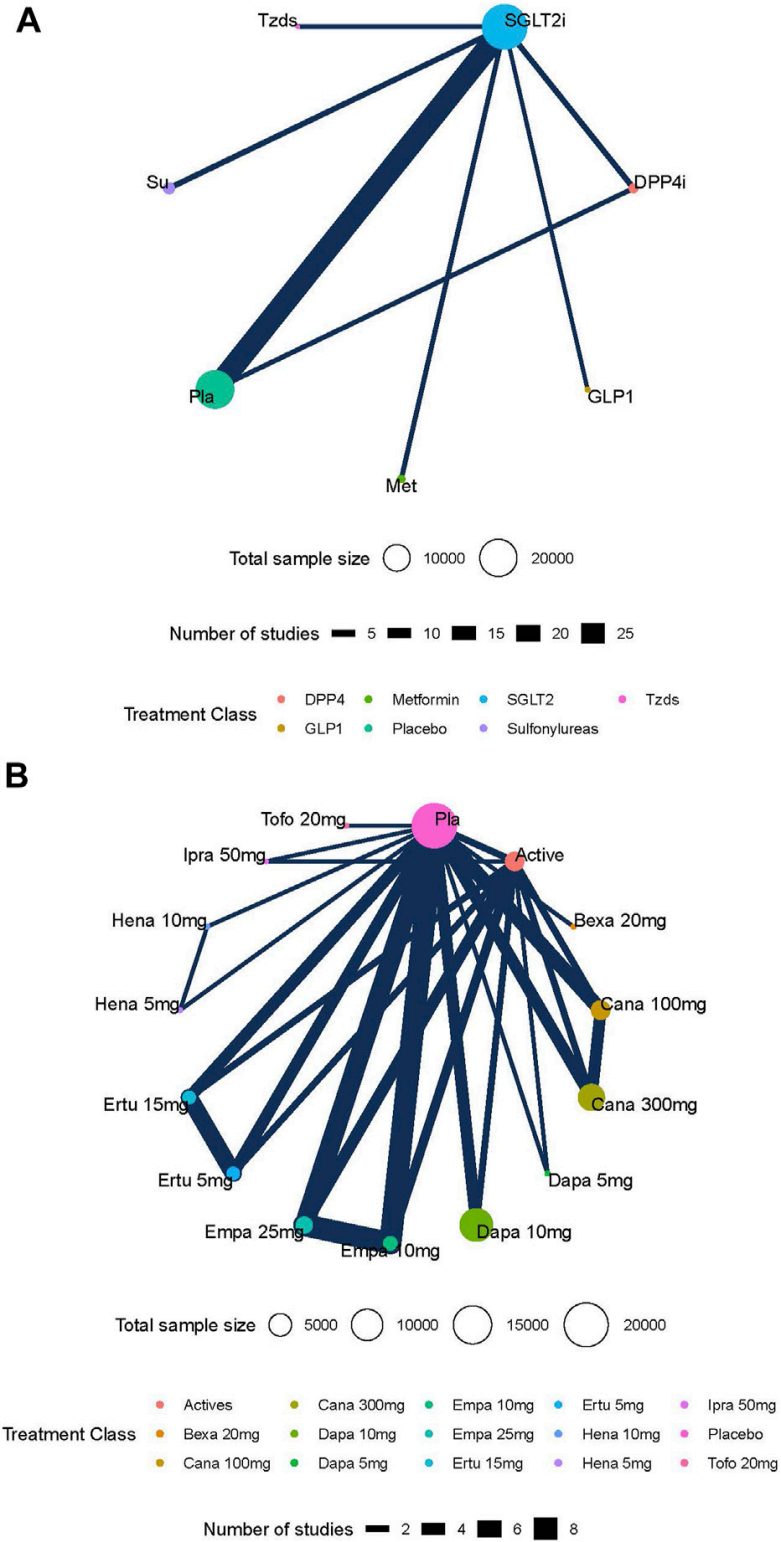
**(A)** Network plots of the risk of diabetic ketoacidosis (DKA) with different kinds of active antidiabetic drugs. **Footnotes:** nodes in different colors indicate different processing. The node size corresponds to the number of participants treated in the study. The thickness of the edge represents the number of tests. The lack of lines suggests that there have been no head-to-head trials of this outcome between the two treatments. **(B)** Network plots of the risk of diabetic ketoacidosis (DKA) with different kinds of active antidiabetic drugs. **Footnotes:** nodes in different colors indicate different processing. The node size corresponds to the number of participants treated in the study. The thickness of the edge represents the number of tests. The lack of lines suggests that there have been no head-to-head trials of this outcome between the two treatment procedures.

The network plots of each outcome are presented in Figure 2A and Figure 2B, presenting the results and quality of evidence for the different doses of SGLT2 inhibitors and the different active antidiabetic drugs. The inconsistency of the network meta-analysis is also evaluated in Supplementary Figure S1 and Supplementary Figure S2. Heterogeneity and intransitivity of the network meta-analysis were also evaluated (Supplementary Table S5–6, Supplementary Figures S3–S5).

### 3.4 DKA

**FIGURE 3:**
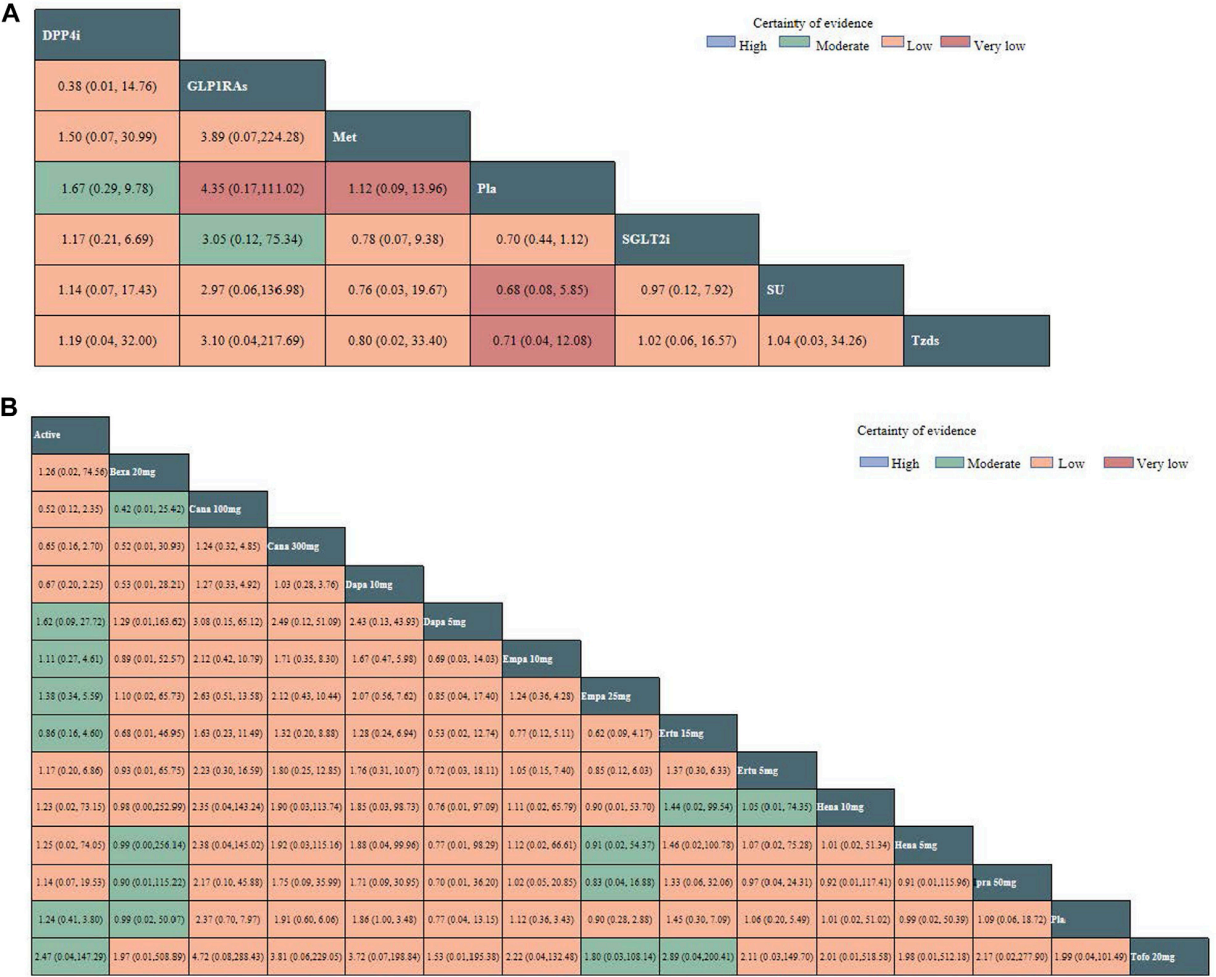
**(A)** Network estimates (league tables) for different kinds of active antidiabetic drugs. **Footnotes:** outcome: the risk of DKA (odds ratio; 95% confidence interval). The league table presented the relative effects of different kinds of active antidiabetic drugs (the risk of DKA on the column to the risk of DKA of the row). DPP4i: dipeptidyl peptidase-4 inhibitor; SGLT2i: sodium–glucose cotransporter 2 inhibitors; SU: sulphonylurea; Tzds: thiazolidinediones; Met: metformin; Pla: placebo; DKA: diabetic ketoacidosis. **(B)** Network estimates (league tables) for different doses of SGLT-2i. **Footnotes:** outcome: the risk of DKA (odds ratio; 95% confidence interval). The league table presented the relative effects of different kinds of SGLT2 inhibitors (the risk of DKA on the column to the risk of DKA of the row). SGLT2i: sodium–glucose cotransporter 2 inhibitors; Cana: canagliflozin; Dapa: dapagliflozin; Ertu: ertugliflozin; Ipra: ipragliflozin; Hena: henagliflozin; Bexa: bexagliflozin; Empa: empagliflozin; Tofo: tofogliflozin; Pla: placebo; DKA: diabetic ketoacidosis.

In total, 36 studies including 52,264 patients were reported on the risk of DKA, with a total of 70 DKA events occurring at a rate of 0.13%. Intervention nodes included in this network meta-analysis were different doses of SGLT2 inhibitors, metformin, sitagliptin, glimepiride, pioglitazone, and placebo. The SGLT2 inhibitor was not associated with a statistically significant increase in the risk of DKA (Figure 3A). There was also no difference in the risk of DKA between different doses of SGLT2 inhibitors (Figure 3B). The global I^2^ of pairwise was 0%, and the global *I* ^*2*^ of the consistency model was 0%. The node split analysis showed that the results were consistent. The GRADE quality for the network meta-analysis is shown in Supplementary Table S7 and Supplementary Table S8.

### 3.5 Rankings and P-score

The P-score of DKA for different kinds of active antidiabetic drugs is illustrated in Supplementary Table S9, and the P-score of DKA for different doses of SGLT-2 inhibitors is illustrated in Supplementary Table S10. A higher P-score indicated a higher risk of DKA. There were no significant differences in network estimates between different hypoglycemic agents, but the P-score of DKA suggested that different kinds of active antidiabetic drugs ranked for the risk of DKA. Supplementary Table S9 shows that the highest P-score was of glucagon-like peptide-1 agonists (GLP1RAs) (P-score = 0.7361). The SGLT2 inhibitor ranked the third (P-score = 0.5298). Likewise, Supplementary Table S10 shows that canagliflozin (100 mg) had the highest P-score (P-score = 0.7388), and the lowest was tofogliflozin (20 mg) (P-score = 0.3491). The P-score was not found to be dose-dependent.

### 3.6 Funnel plot and sensitivity analysis

Egger’s test showed that there is no publication bias for different doses of SGLT2 inhibitors (*p* = 0.10, Supplementary Figure S6), but there is a publication bias for different kinds of active antidiabetic drugs (*p* < 0.01, Supplementary Figure S7). The sensitivity analyses are presented in Supplementary Table S11 and Supplementary Table S12. Studies with a duration of less than 24 weeks were excluded in the sensitivity analysis. The results showed that all sensitivity analyses demonstrated consistency with the primary results, regardless of the inclusion or exclusion of studies lasting less than 24 weeks.

## 4 Discussion

This network meta-analysis provides an overview of the evidence regarding the DKA safety of SGLT2 inhibitors and antidiabetic drugs in patients with T2DM. This result indicated that there was no significant difference in the risk of DKA between different kinds of antidiabetic drugs or different doses of SGLT2 inhibitors with very low-to-moderate certainty. There was no dose-dependent relation between SGLT2 inhibitors and the risk of DKA. Neither antidiabetic drugs nor SGLT2 inhibitors increased the risk of DKA compared with placebo in patients with T2DM. Nevertheless, although not statistically significant, our results indicate that the SGLT2 inhibitor ranked third in the risk of DKA, behind only the GLP-1RA and DPP4 inhibitor. According to the results of ranking and P-score, the risk of DKA slightly elevated among the different kinds of active antidiabetic drugs for glucagon-like peptide 1 receptor agonists (the type of GLP-1RAs included exenatide). Among the different doses of SGLT2 inhibitors, canagliflozin showed a slightly higher risk of DKA, with tofogliflozin being the lowest risk. According to the ranking and P-score, it is found that compared to the high dose, the incidence of DKA with low-dose canagliflozin is even lower.

There have been some DKA case reports linked to the use of canagliflozin, but the exact cause remains to be determined (Turner et al., 2016; Sloan et al., 2018). The potential causes of DKA in patients taking canagliflozin are believed to be four mechanisms: first, fluid loss; second, glucagon secretion increases; third, elevated glucagon–insulin ratio; and finally, acute prerenal azotemia (Chai et al., 2017).

The FDA issued a warning that the use of SGLT2 inhibitors in type 2 diabetes may cause euglycemia DKA (eDKA) in 2015 (FDA, 2015b). The American Diabetes Association also indicated that all patients on SGLT2 inhibitors were at risk for DKA but was rare in T2DM (Draznin et al., 2022). SGLT2 inhibitors may increase the risk of DKA through three possible mechanisms. First, SGLT2 inhibitors lower blood glucose concentrations and increase urinary glucose excretion, which reduces insulin production and promote glucagon production. The lack of insulin leads to the release of α-glycerol from liberals and amino acids from muscle decomposition, which promotes gluconeogenesis and leads to the increase of ketone bodies in the body. Decreasing insulin levels promote the process of lipolysis, which leads to the accumulation of ketones in the body. Second, SGLT2 inhibitors promote ketone body reabsorption by increasing the concentration of sodium ions in the renal tubules. Finally, SGLT2 inhibitors have a diuretic effect and reduce blood volume, thereby promoting the development of ketoacidosis (Cohen et al., 1956; Vallon et al., 2014; Yokono et al., 2014; Mudaliar et al., 2015; Fleming et al., 2020). However, not all patients on SGLT2 inhibitors are at a high risk of DKA. Other factors, such as infection, recent surgery, serious illness, insufficient insulin supply, low-carbohydrate diet, past pancreatitis, and dehydration, can interact with the use of SGLT2 inhibitors and amplify the risk (Bamgboye et al., 2021). Co-action of these risk factors with SGLT2 inhibitors ultimately leads to alter glucose production and increases the production of lipolysis and ketone bodies.

Regarding the use of SGLT2 inhibitors in T2DM, ketoacidosis has been a subject of debate. Different studies have reached inconsistent conclusions about the association between SGLT2 inhibitors and DKA risk. A systematic review including 10 RCTs with a total of 71,553 subjects showed that SGLT2 inhibitors led to increased risks of DKA, and the DKA was approximately three times higher with SGLT2 inhibitors (95%CI 1.36–3.63) (Lin et al., 2021). On the contrary, one unpublished registered clinical trial (NCT03764631) reported that there was no difference in the risk of DKA between empagliflozin and DPP4 inhibitors (Mansour, 2022).

Our analysis confirms the safety of SGLT2 inhibitors with regards to the risk of DKA, similar to that of placebo. In 2019, the United Kingdom Medicines and Health Products Regulatory Agency (MHRA) issued a warning that the use of GLP1RAs in combination with insulin may increase the risk of DKA (MHRA, 2019). This warning is consistent with the conclusions reached in our meta-analysis.

### 4.1 Strengths and limitations

Our study is the first frequentist network meta-analysis of SGLT2 inhibitors investigating the risk of DKA; in addition, we included one unpublished trial from the ClinicalTrials database that provided additional DKA data. Apart from that, we performed the quality assessment on all the included literature studies, ensuring that the literature studies were of high quality. Our systematic review and network meta-analysis included a large pool of trials and patients retrieved through a comprehensive literature search, and we used up-to-date and rigorous methodological tools to assess the risk of bias for the outcome. We included clinical trials from inception through 26 January 2022, with access to additional long-term trials (19 long-term follow-up of 52 weeks or more) and recent studies. A major strength of this network meta-analysis was to compare the risk of DKA among different active antidiabetic drugs. Furthermore, our study supports the evidence of the risk of DKA among different doses of SGLT2 inhibitors, which was the first time to be reported.

Several potential limitations should be acknowledged. First of all, the duration of treatment varied widely among the included studies, ranging from 12 weeks to 271 weeks. Second, because the incidence of DKA was low, the included trials reported a relatively small number of DKA, even reported no DKA event, which caused too much sparse data. We assessed the sparse network with a fixed-effect model and used the sensitivity analysis which excluded the sparse data’ studies for reducing the impact of sparse data. Third, all included studies did not distinguish eDKA and DKA. Therefore, our outcome was DKA not eDKA. Fourth, due to the influence of data collection, the results of our meta-analysis are more relevant to a population that is predominantly male, white, older, longer duration of diabetes, higher body mass index (BMI), and higher HbA1c levels. Moreover, we did not conduct a subgroup analysis to explore the correlation between the risk of SGLT2i-associated DKA and the duration of diabetes because of the range of data variations. There were few studies about the relationship between DKA and the duration of T2DM. An analysis of data on the incidence of DKA in SGLT2 inhibitors from the FDA Adverse Event Reporting System examined that the range of duration was from 1 day to >8 years, and there was no significance due to the limited availability of treatment duration information in some reports (Fadini et al., 2017).

## 5 Conclusion

This network meta-analysis suggested that neither SGLT2 inhibitors nor other active antidiabetic drugs increase the risk of DKA compared with placebo. At the same time, a consistent dose-effect gradient with increasing SGLT2 inhibitors doses was observed for treatment effect but not for the risk of DKA. However, given that the P-score of SGLT2 inhibitors was higher than placebo, SGLT2 inhibitors still needed to be cautiously used for patients with T2DM with a history of DKA. Furthermore, it is better to avoid the use of canagliflozin compared with other SGLT2 inhibitors based on the ranking and P-score. In the future, with the increasing use of SGLT-2 inhibitors, it is crucial to enhance and improve the safety studies of SGLT2 inhibitors by incorporating more clinical trials with large sample sizes and high quality.

## Data Availability

The original contributions presented in the study are included in the article/Supplementary Material, further inquiries can be directed to the corresponding authors.
