# Supplementary material for: Risk of diabetic ketoacidosis of SGLT2 inhibitors in patients with type 2 diabetes: a systematic review and network meta-analysis of randomized controlled trials
Source: Front Pharmacol. 2023 Jun 13;14:1145587. doi: 10.3389/fphar.2023.1145587 (PMC10311413; doi:10.3389/fphar.2023.1145587)

***Supplementary Material***

**Risk of diabetic ketoacidosis of SGLT2 inhibitors in patients with type 2 diabetes: a systematic review and network meta-analysis of randomised controlled trials**

**Shiwen Yang^†^, Yin Liu^†^, Shengzhao Zhang^†^, Fengbo Wu, Dan Liu, Qingfang Wu, Hanrui Zheng, Ping Fan*, Na Su***

*** Correspondence:** Na Su: [zoya159@163.com](mailto:zoya159@163.com). Ping Fan: 825370320@qq.com.

## Supplementary Table 1. PRISMA Checklist. PRISMA checklist for this network meta-analysis

| **Section/topic** | **#** | **Checklist item** | **Reported on page #** |
| --- | --- | --- | --- |
| **TITLE** | | |  |
| Title | 1 | Identify the report as a systematic review incorporating a network meta-analysis (or related form of meta-analysis). | 1 |
| **ABSTRACT** | | |  |
| Structured summary | 2 | Provide a structured summary including, as applicable: Background: main objectives  Methods: data sources; study eligibility criteria, participants, and interventions; study appraisal; and synthesis methods, such as network meta-analysis.  Results: number of studies and participants identified; summary estimates with corresponding confidence/credible intervals; treatment rankings may also be discussed. Authors may choose to summarize pairwise comparisons against a chosen treatment included in their analyses for brevity.  Discussion/Conclusions: limitations; conclusions and implications of findings.  Other: primary source of funding; systematic review registration number with registry name. | 2, 3 |
| **INTRODUCTION** | | |  |
| Rationale | 3 | Describe the rationale for the review in the context of what is already known, including mention of why a network meta-analysis has been conducted. | 4 |
| Objectives | 4 | Provide an explicit statement of questions being addressed with reference to participants, interventions, comparisons, outcomes, and study design (PICOS). | 4 |
| **METHODS** | | |  |
| Protocol and registration | 5 | Indicate if a review protocol exists, if and where it can be accessed (e.g., Web address), and, if available, provide registration information including registration number. | 5 |
| Eligibility criteria | 6 | Specify study characteristics (e.g., PICOS, length of follow-up) and report characteristics (e.g., years considered, language, publication status) used as criteria for eligibility, giving rationale.  Clearly describe eligible treatments included in the treatment network, and note whether any have been clustered or merged into the same node (with justification). | 5 |
| Information sources | 7 | Describe all information sources (e.g., databases with dates of coverage, contact with study authors to identify additional studies) in the search and date last searched. | 5, 6 |
| Search | 8 | Present full electronic search strategy for at least one database, including any limits used, such that it could be repeated. | 5, 6 |
| Study selection | 9 | State the process for selecting studies (i.e., screening, eligibility, included in systematic review, and, if applicable, included in the meta-analysis). | 5, 6 |
| Data collection process | 10 | Describe method of data extraction from reports (e.g., piloted forms, independently, in duplicate) and any processes for obtaining and confirming data from investigators. | 6 |
| Data items | 11 | List and define all variables for which data were sought (e.g., PICOS, funding sources) and any assumptions and simplifications made. | 7 |
| Risk of bias in individual studies | 12 | Describe methods used for assessing risk of bias of individual studies (including specification of whether this was done at the study or outcome level), and how this information is to be used in any data synthesis. | 7 |
| Summary measures | 13 | State the principal summary measures (e.g., risk ratio, difference in means). Also describe the use of additional summary measures assessed, such as treatment rankings and surface under the cumulative ranking curve (SUCRA) values, as well as modified approaches used to present summary findings from meta-analyses. | 7 |
| Planned methods of analysis | 14 | Describe the methods of handling data and combining results of studies for each network meta-analysis. This should include, but not be limited to:  Handling of multigroup trials;  Selection of variance structure;  Selection of prior distributions in Bayesian analyses; and  Assessment of model fit. | 7 |
| Assessment of inconsistency | S2 | Describe the statistical methods used to evaluate the agreement of direct and indirect evidence in the treatment network(s) studied. Describe efforts taken to address its presence when found. | 7 |
| Risk of bias across studies | 15 | Specify any assessment of risk of bias that may affect the cumulative evidence (e.g., publication bias, selective reporting within studies). | 7 |
| Additional analyses | 16 | Describe methods of additional analyses if done, indicating which were prespecified. This may include, but not be limited to, the following:  Sensitivity or subgroup analyses;  Meta-regression analyses;  Alternative formulations of the treatment network; and  Use of alternative prior distributions for Bayesian analyses (if applicable). | 7 |
| **RESULTS** |  |  |  |
| Study selection | 17 | Give numbers of studies screened, assessed for eligibility, and included in the review, with reasons for exclusions at each stage, ideally with a flow diagram. | 8, Figure 1 |
| Presentation of network structure | S3 | Provide a network graph of the included studies to enable visualization of the geometry of the treatment network. | Figure 1 |
| Summary of network geometry | S4 | Provide a brief overview of characteristics of the treatment network. This may include commentary on the abundance of trials and randomized patients for the different interventions and pairwise comparisons in the network, gaps of evidence in the treatment network, and potential biases reflected by the network structure. | 7, 8 |
| Study characteristics | 18 | For each study, present characteristics for which data were extracted (e.g., study size, PICOS, follow-up period) and provide the citations. | 7, 8  Table 1 |
| Risk of bias within studies | 19 | Present data on risk of bias of each study and, if available, any outcome level assessment. | 8 |
| Results of individual studies | 20 | For all outcomes considered (benefits or harms), present, for each study: 1) simple summary data for each intervention group, and 2) effect estimates and confidence intervals. Modified approaches may be needed to deal with information from larger networks. | 9 |
| Synthesis of results | 21 | Present results of each meta-analysis done, including confidence/credible intervals. In larger networks, authors may focus on comparisons versus a particular comparator (e.g., placebo or standard care), with full findings presented in an appendix. League tables and forest plots may be considered to summarize pairwise comparisons. If additional summary measures were explored (such as treatment rankings), these should also be presented. | 9, 10  Table 2a  Table 2b |
| Exploration for inconsistency | S5 | Describe results from investigations of inconsistency. This may include such information as measures of model fit to compare consistency and inconsistency models, P values from statistical tests, or summary of inconsistency estimates from different parts of the treatment network. | 9, 10 |
| Risk of bias across studies | 22 | Present results of any assessment of risk of bias across studies (see Item 15). | 9, 10 |
| Additional analysis | 23 | Give results of additional analyses, if done (e.g., sensitivity or subgroup analyses, meta-regression analyses, alternative network geometries studied, alternative choice of prior distributions for Bayesian analyses, and so forth). | 10 |
| **DISCUSSION** |  |  |  |
| Summary of evidence | 24 | Summarize the main findings including the strength of evidence for each main outcome; consider their relevance to key groups (e.g., healthcare providers, users, and policy makers). | 10, 11, 12 |
| Limitations | 25 | Discuss limitations at study and outcome level (e.g., risk of bias), and at review-level (e.g., incomplete retrieval of identified research, reporting bias). | 12 |
| Conclusions | 26 | Provide a general interpretation of the results in the context of other evidence, and implications for future research. | 13 |
| **FUNDING** |  |  |  |
| Funding | 27 | Describe sources of funding for the systematic review and other support (e.g., supply of data); role of funders for the systematic review. | 13 |

##

## Supplementary Table 2. Search strategy

| Database | Search strategy |
| --- | --- |
| PubMed | Search: ((ketoacidosis[tw] OR ketoacidosis[mh])) AND (((((sglt [tw]) OR ((sglt2 [tw]) OR (Sodium-Glucose Transport Proteins [mh]))) OR ((((((((((((((((((((((((((((((gliflozins[mh]) OR (gliflozins[tw])) OR (bexagliflozin[mh])) OR (bexagliflozin[tw])) OR (canagliflozin[mh])) OR (canagliflozin[tw])) OR (dapagliflozin[mh])) OR (dapagliflozin[tw])) OR (empagliflozin[mh])) OR (empagliflozin[tw])) OR (ertugliflozin[mh])) OR (ertugliflozin[tw])) OR (henagliflozin[mh])) OR (henagliflozin[tw])) OR (ipragliflozin[mh])) OR (ipragliflozin[tw])) OR (licogliflozin[mh])) OR (licogliflozin[tw])) OR (luseogliflozin[mh])) OR (luseogliflozin[tw])) OR (remogliflozins[mh])) OR (remogliflozins[tw])) OR (sergliflozin[mh])) OR (sergliflozin[tw])) OR (sotagliflozin[mh])) OR (sotagliflozin[tw])) OR (tofogliflozin[mh])) OR (tofogliflozin[tw])) OR (gliflozins[mh])) OR (gliflozins[tw]))) AND (Diabetes Mellitus, Type 2 [mh])) AND (randomized controlled trial[pt] OR controlled clinical trial[pt] OR clinical trials as topic [mesh: noexp] OR randomized[tiab] OR randomised [tiab] OR placebo [tiab] OR randomly [tiab] OR trial [ti] OR drug therapy [sh] OR groups [tiab])) |
| Embase (Ovid SP) | (exp non insulin dependent diabetes mellitus/ OR (Diabetes Mellitus, Type 2 or Type 2 Diabetes Mellitus or niddm* or mody* or DMT2 or T2DM or stable diabete*).mp.) AND (exp sodium glucose cotransporter 2/ or exp sodium glucose cotransporter 2 inhibitor/ or sglt.mp. or sglt-2.mp. or sglt2.mp. or Sodium-Glucose Transport Protein$.mp. or Sodium-glucose transporter$.mp. or Sodium-glucose co-transporter$.mp. or Sodium glucose cotransporter$.mp. or gliflozins.mp. or exp gliflozins/ or bexagliflozin.mp. or exp bexagliflozin/ or canagliflozin.mp. or exp canagliflozin/ or dapagliflozin.mp. or exp dapagliflozin/ or empagliflozin.mp. or exp empagliflozin/ or ertugliflozin.mp. or exp ertugliflozin/ or henagliflozin.mp. or exp henagliflozin/ or ipragliflozin.mp. or exp ipragliflozin/ or licogliflozin.mp. or exp licogliflozin/ or luseogliflozin.mp. or exp luseogliflozin/ or remogliflozin.mp. or exp remogliflozin etabonate/ or sergliflozin.mp. or exp sergliflozin etabonate/ or sotagliflozin.mp. or exp sotagliflozin/ or tofogliflozin.mp. or exp tofogliflozin/ or gliflozin.mp. or exp gliflozin/) AND (Clinical trial/ or Randomized controlled trial/ or Randomization/ or Randomi?ed controlled trial$.tw. or Rct.tw. or Random allocation.tw. or Randomly allocated.tw. or Allocated randomly.tw. or (allocated adj2 random).tw.) AND (exp ketoacidosis/ or ketoacidosis.mp.) |
| Cochrane Central Register of Controlled Trials (via OVID) | (exp Diabetes Mellitus, Type 2/ OR (Diabetes Mellitus, Type 2 or Type 2 Diabetes Mellitus or niddm* or mody* or DMT2 or T2DM or stable diabete*).mp.) AND (exp sodium glucose cotransporter 2/ or exp sodium glucose cotransporter 2 inhibitor/ or sglt.mp. or sglt-2.mp. or sglt2.mp. or Sodium-Glucose Transport Protein$.mp. or Sodium-glucose transporter$.mp. or Sodium-glucose co-transporter$.mp. or Sodium glucose cotransporter$.mp. or gliflozins.mp. or exp gliflozins/ or bexagliflozin.mp. or exp bexagliflozin/ or canagliflozin.mp. or exp canagliflozin/ or dapagliflozin.mp. or exp dapagliflozin/ or empagliflozin.mp. or exp empagliflozin/ or ertugliflozin.mp. or exp ertugliflozin/ or henagliflozin.mp. or exp henagliflozin/ or ipragliflozin.mp. or exp ipragliflozin/ or licogliflozin.mp. or exp licogliflozin/ or luseogliflozin.mp. or exp luseogliflozin/ or remogliflozin.mp. or exp remogliflozin etabonate/ or sergliflozin.mp. or exp sergliflozin etabonate/ or sotagliflozin.mp. or exp sotagliflozin/ or tofogliflozin.mp. or exp tofogliflozin/ or gliflozin.mp. or exp gliflozin/) AND (Clinical trial/ or Randomized controlled trial/ or Randomization/ or Randomi?ed controlled trial$.tw. or Rct.tw. or Random allocation.tw. or Randomly allocated.tw. or Allocated randomly.tw. or (allocated adj2 random).tw.) |

## Supplementary included research

Allegretti, A. S., Zhang, W., Zhou, W., Thurber, T. K., Rigby, S. P., Bowman-Stroud, C., et al. (2019). Safety and Effectiveness of Bexagliflozin in Patients With Type 2 Diabetes Mellitus and Stage 3a/3b CKD. *Am J Kidney Dis*. 74(3), 328-337.

Araki, E., Onishi, Y., Asano, M., Kim, H. & Yajima, T. (2017). Efficacy and safety of dapagliflozin over 1 year as add-on to insulin therapy in Japanese patients with type 2 diabetes: the DAISY (Dapagliflozin Added to patients under InSulin therapY) trial. *Diabetes Obes Metab*. 19(4), 562-570.

Aronson, R., Frias, J., Goldman, A., Darekar, A., Lauring, B. & Terra, S. G. (2018). Long-term efficacy and safety of ertugliflozin monotherapy in patients with inadequately controlled T2DM despite diet and exercise: VERTIS MONO extension study. *Diabetes Obes Metab*. 20(6), 1453-1460.

Barnett, A. H., Mithal, A., Manassie, J., Jones, R., Rattunde, H., Woerle, H. J., et al. (2014). Efficacy and safety of empagliflozin added to existing antidiabetes treatment in patients with type 2 diabetes and chronic kidney disease: a randomised, double-blind, placebo-controlled trial. *The Lancet Diabetes & Endocrinology*. 2(5), 369-384.

Bode, B., Stenlof, K., Sullivan, D., Fung, A. & Usiskin, K. (2013). Efficacy and safety of canagliflozin treatment in older subjects with type 2 diabetes mellitus: a randomized trial. *Hosp Pract (1995)*. 41(2), 72-84.

Cahn, A., Raz, I., Bonaca, M., Mosenzon, O., Murphy, S. A., Yanuv, I., et al. (2020). Safety of dapagliflozin in a broad population of patients with type 2 diabetes: Analyses from the DECLARE-TIMI 58 study. *Diabetes Obes Metab*. 22(8), 1357-1368.

Cho, K. Y., Nakamura, A., Omori, K., Takase, T., Miya, A., Manda, N., et al. (2019). Effect of switching from pioglitazone to the sodium glucose co-transporter-2 inhibitor dapagliflozin on body weight and metabolism-related factors in patients with type 2 diabetes mellitus: An open-label, prospective, randomized, parallel-group comparison trial. *Diabetes Obes Metab*. 21(3), 710-714.

Dagogo-Jack, S., Liu, J., Eldor, R., Amorin, G., Johnson, J., Hille, D., et al. (2018). Efficacy and safety of the addition of ertugliflozin in patients with type 2 diabetes mellitus inadequately controlled with metformin and sitagliptin: The VERTIS SITA2 placebo-controlled randomized study. *Diabetes Obes Metab*. 20(3), 530-540.

Fioretto, P., Del Prato, S., Buse, J. B., Goldenberg, R., Giorgino, F., Reyner, D., et al. (2018). Efficacy and safety of dapagliflozin in patients with type 2 diabetes and moderate renal impairment (chronic kidney disease stage 3A): The DERIVE Study. *Diabetes Obes Metab*. 20(11), 2532-2540.

Frías, J. P., Guja, C., Hardy, E., Ahmed, A., Dong, F., Öhman, P., et al. (2016). Exenatide once weekly plus dapagliflozin once daily versus exenatide or dapagliflozin alone in patients with type 2 diabetes inadequately controlled with metformin monotherapy (DURATION-8): a 28 week, multicentre, double-blind, phase 3, randomised controlled trial. *The Lancet Diabetes & Endocrinology*, 4(12), 1004-1016.

Hadjadj S, R. J., Meinicke T, Woerle HJ, Broedl UC (2016) Initial Combination of Empagliflozin and Metformin in Patients With Type 2 Diabetes. *Diabetes Care*. 39(10), 1718-1728.

Haering, H. U., Merker, L., Christiansen, A. V., Roux, F., Salsali, A., Kim, G., et al. (2015). Empagliflozin as add-on to metformin plus sulphonylurea in patients with type 2 diabetes. *Diabetes Res Clin Pract*. 110(1), 82-90.

Han, K. A., Chon, S., Chung, C. H., Lim, S., Lee, K. W., Baik, S., et al. (2018). Efficacy and safety of ipragliflozin as an add-on therapy to sitagliptin and metformin in Korean patients with inadequately controlled type 2 diabetes mellitus: A randomized controlled trial. *Diabetes Obes Metab*. 20(10), 2408-2415.

Hollander, P., Liu, J., Hill, J., Johnson, J., Jiang, Z. W., Golm, G., et al (2018) Ertugliflozin Compared with Glimepiride in Patients with Type 2 Diabetes Mellitus Inadequately Controlled on Metformin: The VERTIS SU Randomized Study. *Diabetes Ther*, 9(1), 193-207.

Ito D, S. S., Inoue K, Saito D, Yanagisawa M, Inukai K, Akiyama Y, Morimoto Y, Noda M, Shimada A. (2017). Comparison of Ipragliflozin and Pioglitazone Effects on Nonalcoholic Fatty Liver Disease in Patients With Type 2 Diabetes: A Randomized, 24-Week, Open-Label, Active-Controlled Trial. *Diabetes Care*. 40(10), 1364-1372.

Ji, L., Liu, Y., Miao, H., Xie, Y., Yang, M., Wang, W., et al. (2019). Safety and efficacy of ertugliflozin in Asian patients with type 2 diabetes mellitus inadequately controlled with metformin monotherapy: VERTIS Asia. *Diabetes Obes Metab*. 21(6), 1474-1482.

Kawamori, R., Haneda, M., Suzaki, K., Cheng, G., Shiki, K., Miyamoto, Y., et al. (2018). Empagliflozin as add-on to linagliptin in a fixed-dose combination in Japanese patients with type 2 diabetes: Glycaemic efficacy and safety profile in a 52-week, randomized, placebo-controlled trial. *Diabetes Obes Metab*. 20(9), 2200-2209.

Lavalle-González FJ, J. A., Davidson J, Tong C, Qiu R, Canovatchel W, Meininger G. (2013). Efficacy and safety of canagliflozin compared with placebo and sitagliptin in patients with type 2 diabetes on background metformin monotherapy: a randomised trial. *Diabetologia*, 56(12), 2582-2592.

Mancia G, C. C., Tikkanen I, Zeller C, Ley L, Woerle HJ, Broedl UC, Johansen OE. (2016). Impact of Empagliflozin on Blood Pressure in Patients With Type 2 Diabetes Mellitus and Hypertension by Background Antihypertensive Medication. *Hypertension*. 68(6), 1355-1364.

Neal, B., Perkovic, V., Mahaffey, K. W., de Zeeuw, D., Fulcher, G., Erondu, N., et al. (2017). Canagliflozin and Cardiovascular and Renal Events in Type 2 Diabetes. *N Engl J Med*. 377(7), 644-657.

Perkovic, V., Jardine, M. J., Neal, B., Bompoint, S., Heerspink, H. J. L., Charytan, D. M., et al. (2019). Canagliflozin and Renal Outcomes in Type 2 Diabetes and Nephropathy. *New England Journal of Medicine*. 380(24), 2295-2306.

Persson F, R. P., Vart P, Chertow GM, Hou FF, Jongs N, McMurray JJV, Correa-Rotter R, Bajaj HS, Stefansson BV, Toto RD, Langkilde AM, Wheeler DC, Heerspink HJL; DAPA-CKD Trial Committees and Investigators. (2021). Efficacy and Safety of Dapagliflozin by Baseline Glycemic Status: A Prespecified Analysis From the DAPA-CKD Trial. *Diabetes Care*. 44(8), 1894-1897.

Pollock, C., Stefánsson, B., Reyner, D., Rossing, P., Sjöström, C. D., Wheeler, D. C., et al. (2019). Albuminuria-lowering effect of dapagliflozin alone and in combination with saxagliptin and effect of dapagliflozin and saxagliptin on glycaemic control in patients with type 2 diabetes and chronic kidney disease (DELIGHT): a randomised, double-blind, placebo-controlled trial. *The Lancet Diabetes & Endocrinology*, 7(6), 429-441.

Pratley, R. E., Eldor, R., Raji, A., Golm, G., Huyck, S. B., Qiu, Y., et al. (2018). Ertugliflozin plus sitagliptin versus either individual agent over 52 weeks in patients with type 2 diabetes mellitus inadequately controlled with metformin: The VERTIS FACTORIAL randomized trial. *Diabetes Obes Metab*. 20(5), 1111-1120.

Ridderstrale, M., Rosenstock, J., Andersen, K. R., Woerle, H. J., Salsali, A. & investigators, E.-R. H. H. S. t. (2018). Empagliflozin compared with glimepiride in metformin-treated patients with type 2 diabetes: 208-week data from a masked randomized controlled trial. *Diabetes Obes Metab*. 20(12), 2768-2777.

Rodbard, H. W., Seufert, J., Aggarwal, N., Cao, A., Fung, A., Pfeifer, M., et al. (2016). Efficacy and safety of titrated canagliflozin in patients with type 2 diabetes mellitus inadequately controlled on metformin and sitagliptin. *Diabetes Obes Metab*. 18(8), 812-9.

Roden, M., Merker, L., Christiansen, A. V., Roux, F., Salsali, A., Kim, G., et al. (2015). Safety, tolerability and effects on cardiometabolic risk factors of empagliflozin monotherapy in drug-naive patients with type 2 diabetes: a double-blind extension of a Phase III randomized controlled trial. *Cardiovasc Diabetol*. 14, 154.

Rosenstock J, C. L., González-Ortiz M, Merton K, Craig J, Capuano G, Qiu R. (2016). Initial Combination Therapy With Canagliflozin Plus Metformin Versus Each Component as Monotherapy for Drug-Naïve Type 2 Diabetes. *Diabetes Care*. 39(3), 353-362.

Rosenstock, J., Frias, J., Pall, D., Charbonnel, B., Pascu, R., Saur, D., et al. (2018). Effect of ertugliflozin on glucose control, body weight, blood pressure and bone density in type 2 diabetes mellitus inadequately controlled on metformin monotherapy (VERTIS MET). *Diabetes Obes Metab*. 20(3), 520-529.

Rosenstock J, J. A., Frappin G, Salsali A, Kim G, Woerle HJ, Broedl UC. (2014). EMPA-REG MDI Trial Investigators. Improved glucose control with weight loss, lower insulin doses, and no increased hypoglycemia with empagliflozin added to titrated multiple daily injections of insulin in obese inadequately controlled type 2 diabetes. *Diabetes Care*. 37(7), 1815-1823.

Rosenstock, J., Jelaska, A., Zeller, C., Kim, G., Broedl, U. C., Woerle, H. J., et al. (2015). Impact of empagliflozin added on to basal insulin in type 2 diabetes inadequately controlled on basal insulin: a 78-week randomized, double-blind, placebo-controlled trial. *Diabetes Obes Metab*, 17(10), 936-48.

Scott, R., Morgan, J., Zimmer, Z., Lam, R. L. H., O'Neill, E. A., Kaufman, K. D., et al. (2018). A randomized clinical trial of the efficacy and safety of sitagliptin compared with dapagliflozin in patients with type 2 diabetes mellitus and mild renal insufficiency: The CompoSIT-R study. *Diabetes Obes Metab*. 20(12), 2876-2884.

Søfteland E, M. J., Vangen B, Toorawa R, Maldonado-Lutomirsky M, Broedl UC. (2017). Empagliflozin as Add-on Therapy in Patients With Type 2 Diabetes Inadequately Controlled With Linagliptin and Metformin: A 24-Week Randomized, Double-Blind, Parallel-Group Trial. *Diabetes Care*. 40(2), 201-209.

Terauchi, Y., Tamura, M., Senda, M., Gunji, R. & Kaku, K. (2018). Long-term safety and efficacy of tofogliflozin as add-on to insulin in patients with type 2 diabetes: Results from a 52-week, multicentre, randomized, double-blind, open-label extension, Phase 4 study in Japan (J-STEP/INS). *Diabetes Obes Metab*. 20(5), 1176-1185.

Weng, J., Zeng, L., Zhang, Y., Qu, S., Wang, X., Li, P., et al. (2021). Henagliflozin as add-on therapy to metformin in patients with type 2 diabetes inadequately controlled with metformin: A multicentre, randomized, double-blind, placebo-controlled, phase 3 trial. *Diabetes Obes Metab*. 23(8), 1754-1764.

## Supplementary Table 3. Summary of characteristics of included studies

| \| **Study characteristics** \| **No. (%) / Mean** \| **IQR** \| **Range** \| \| --- \| --- \| --- \| --- \| \| **Eligible studies:** \|  \|  \|  \| \| **Total No of trials** \| 36 \|  \|  \| \| **No of participants** \| 52264 \|  \|  \| \| **Duration of diabetes (years)** \| 9.8 \| 7.0 to 12.9 \| 3.3 to 17.7 \| \| **The duration of treatment(weeks)** \| 61.7 \| 24.0 to 66.0 \| 12.0 to 271.0 \| \| **Region:** \|  \|  \|  \| \| **Multinational** \| 25 (69.44%) \|  \|  \| \| **China** \| 2 (5.56%) \|  \|  \| \| **Japan** \| 4 (11.11%) \|  \|  \| \| **Korean** \| 1 (2.78%) \|  \|  \| \| **USA** \| 4 (11.2%) \|  \|  \| \| **Participants:** \|  \|  \|  \| \| **Mean age (years)** \| 59.3 \| 55.7 to 62.9 \| 51.6 to 69.9 \| \| **Male (%)** \| 60.9 \| 51.2 to 63.1 \| 39.9 to 78.0 \| \| **Baseline mean BMI (kg/m^2^)** \| 30.4 \| 28.7 to 32.0 \| 25.4 to 35.0 \| \| **Mean HbA1c (%)** \| 8.1 \| 7.9 to 8.4 \| 6.9 to 9.3 \|   **Footnotes:** IQR: interquartile range; BMI: body mass index; HbA1c: Hemoglobin A1c |
| --- | --- | --- | --- | --- | --- | --- | --- | --- | --- | --- | --- | --- | --- | --- | --- | --- | --- | --- | --- | --- | --- | --- | --- | --- | --- | --- | --- | --- | --- | --- | --- | --- | --- | --- | --- | --- | --- | --- | --- | --- | --- | --- | --- | --- | --- | --- | --- | --- | --- | --- | --- | --- | --- | --- | --- | --- | --- | --- | --- | --- | --- | --- | --- | --- | --- | --- | --- | --- |

## Supplementary Table 4. Risk of bias assessments

| **Study** | **D1** | **D2** | **D3** | **D4** | **D5** | **Overall** |
| --- | --- | --- | --- | --- | --- | --- |
| Andrew 2019 | Low | Some concerns | Low | Low | Low | Some concerns |
| Aronson 2016 | Low | Some concerns | Low | Low | Low | Some concerns |
| Araki 2016 | Low | Some concerns | Low | Low | Low | Some concerns |
| Bode 2013 | Low | Low | Low | Low | Low | Low |
| Barnett 2014 | Low | Low | Low | Low | Low | Low |
| Cahn 2020 | Some concerns | Some concerns | Low | Low | Low | Some concerns |
| Dagogo-Jack 2018 | Low | Low | Low | Low | Low | Low |
| Daisuke It 2017 | Low | Low | Low | Low | Low | Low |
| Frías 2016 | Low | Low | Low | Low | Low | Low |
| Fioretto 2018 | Low | Some concerns | Low | Low | Low | Some concerns |
| Han 2018 | Some concerns | Some concerns | Low | Low | Low | Some concerns |
| Hollander 2018 | Low | Low | Low | Low | Low | Low |
| Haering 2015 | Some concerns | Low | Low | Low | Low | Some concerns |
| Hadiadj 2016 | Low | Some concerns | Low | Low | Low | Some concerns |
| Ji 2019 | Low | Low | Low | Low | Low | Low |
| Kawamori 2018 | Low | Some concerns | Low | Low | Low | Some concerns |
| Kyu Yong Cho 2018 | Some concerns | Some concerns | Low | Low | Low | Some concerns |
| Lavalle-González  2013 | Low | Low | Low | Low | Low | Low |
| NCT01106625 2013 | Some concerns | Some concerns | Low | Low | Low | Some concerns |
| Mancia 2016 | Some concerns | Low | Low | Low | Low | Some concerns |
| Rosenstock 2014 | Low | Low | Low | Low | Low | Low |
| Neal 2017 | Low | Low | Low | Low | Low | Low |
| Perkovic 2019 | Low | Low | Low | Low | Low | Low |
| Persson 2021 | Low | Low | Low | Low | Low | Low |
| Pollock 2019 | Low | Low | Low | Low | Low | Low |
| Pratley 2018 | Low | Low | Low | Low | Low | Low |
| Rodbard 2016 | Low | Low | Low | Low | Low | Low |
| Rosenstock 2018 | Low | Low | Low | Low | Low | Low |
| Roden 2015 | Low | Low | Low | Low | Low | Low |
| Ridderstråle 2018 | Low | Low | Low | Low | Low | Low |
| Rosenstock 2016 | Low | Low | Low | Low | Low | Low |
| Rosenstock 2015 | Low | Low | Low | Low | Low | Low |
| Søfteland 2016 | Low | Some concerns | Low | Low | Low | Some concerns |
| Scott 2018 | Low | Some concerns | Low | Low | Low | Some concerns |
| Terauchi 2017 | Low | Some concerns | Low | Low | Low | Some concerns |
| Weng 2021 | Low | Low | Low | Low | Low | Low |

**Footnote:** D1: Risk of bias arising from the randomization process; D2: Risk of bias due to deviations from the intended interventions; D3: Risk of bias due to missing outcome data; D4: Risk of bias in measurement of the outcome; D5: Risk of bias in selection of the reported result; Overall: Overall risk of bias

## Supplementary Table 5. Heterogeneity assessments for different kinds of active antidiabetic drugs

| **Outcomes** | **Design-based Q statistic, comparisons, and overall statement** | **Q statistic** | **Degree of freedom** | **P value** |
| --- | --- | --- | --- | --- |
| **The risk of DKA** | **Design-specific decomposition of within-designs Q statistic** | | | |
|  | Met vs SGLT-2i | 0.03 | 1 | 0.8741 |
|  | Pla vs SGLT-2i | 13.95 | 24 | 0.9477 |
|  | SGLT-2i vs SU | 0.18 | 2 | 0.9143 |
|  | SGLT-2i vs Tzds | 0.00 | 1 | 0.9755 |
|  | Pla vs DPP4i vs SGLT-2i | 1.66 | 2 | 0.4365 |
|  | **Between-designs Q statistic after detaching of single designs** | | | |
|  | DPP4i vs SGLT-2i | 0.00 | 1 | 0.9547 |
|  | Pla vs SGLT-2i | 0.01 | 1 | 0.9204 |
|  | Pla vs DPP4i vs SGLT-2i | 0.00 | 0 | - |
|  | Q statistic to assess consistency under the assumption of a full design-by-treatment interaction random effects model: between designs Q statistic, 0.01; degree of freedom, 2; p value, 0.9943; tau.within, 0; tau2.within, 0. | | | |

**Footnote:** Q statistic: Cochran’s Q; tau.within: tau statistic within design; DPP4i: Dipeptidyl peptidase-4 inhibitor; SGLT2i: Sodium-glucose cotransporter-2 inhibitors; SU: Sulphonylurea; Tzds: Thiazolidinediones; Met: Metformin; Pla: Placebo; DKA: Diabetic ketoacidosis.

## Supplementary Table 6. Heterogeneity assessments for different doses of SGLT-2i

| **Outcomes** | **Design-based Q statistic, comparisons, and overall statement** | **Q statistic** | **Degree of freedom** | **P value** |
| --- | --- | --- | --- | --- |
| **The risk of DKA** | **Design-specific decomposition of within-designs Q statistic** | | | |
|  | Active vs Dapa 10mg | 0.19 | 1 | 0.6650 |
|  | Pla vs Cana 100mg | 1.14 | 1 | 0.2863 |
|  | Pla vs Dapa 10mg | 2.52 | 3 | 0.4715 |
|  | Active vs Ertu 15mg vs Ertu 5mg | 0.26 | 2 | 0.8777 |
|  | Pla vs Cana 100mg vs Cana 300mg | 0.92 | 2 | 0.6305 |
|  | Pla vs Empa 10mg vs Empa 25mg | 1.02 | 10 | 0.9998 |
|  | Pla vs Ertu 15mg vs Ertu 5mg | 0.00 | 6 | 1.0000 |
|  | **Between-designs Q statistic after detaching of single designs** | | | |
|  | Active vs Dapa 10mg | 6.44 | 17 | 0.9898 |
|  | Active vs Dapa 5mg | 7.28 | 17 | 0.9796 |
|  | Active vs Empa 25mg | 7.35 | 17 | 0.9785 |
|  | Active vs Ipra 50mg | 7.37 | 17 | 0.9783 |
|  | Pla vs Cana 100mg | 5.17 | 17 | 0.9972 |
|  | Pla vs Cana 300mg | 7.24 | 17 | 0.9802 |
|  | Pla vs Dapa 10mg | 6.44 | 17 | 0.9898 |
|  | Pla vs Dapa 5mg | 7.28 | 17 | 0.9796 |
|  | Pla vs Empa 10mg | 7.22 | 17 | 0.9805 |
|  | Pla vs Ipra 50mg | 7.37 | 17 | 0.9783 |
|  | Active vs Cana 100mg vs Cana 300mg | 6.52 | 16 | 0.9814 |
|  | Active vs Empa 10mg vs Empa 25mg | 7.05 | 16 | 0.9722 |
|  | Active vs Ertu 15mg vs Ertu 5mg | 6.98 | 16 | 0.9737 |
|  | Pla vs Active vs Cana 100mg vs Cana 300mg | 5.12 | 15 | 0.9911 |
|  | Pla vs Active vs Empa 10mg vs Empa 25mg | 5.73 | 15 | 0.9839 |
|  | Pla vs Cana 100mg vs Cana 300mg | 7.31 | 16 | 0.9667 |
|  | Pla vs Empa 10mg vs Empa 25mg | 5.54 | 16 | 0.9923 |
|  | Pla vs Ertu 15mg vs Ertu 5mg | 6.98 | 16 | 0.9737 |
|  | Q statistic to assess consistency under the assumption of a full design-by-treatment interaction random effects model: between designs Q statistic, 7.38; degree of freedom, 18; p value, 0.9865; tau.within, 0; tau2.within, 0. | | | |

**Footnote:** Q statistic: Cochran’s Q; tau.within: tau statistic within design; SGLT-2i: Sodium-glucose co-transporter-2 inhibitors; Cana: Canagliflozin; Dapa: Dapagliflozin; Ertu: Ertugliflozin; Ipra: Ipragliflozin; Hena: Henagliflozin; Bexa: Bexagliflozin; Empa: Empagliflozin; Tofo: Tofogliflozin; Pla:Placebo; DKA: diabetic ketoacidosis.

## Supplementary Table 7. GRADE assessments for different kinds of active antidiabetic drugs

| **Comparison** | **Direct estimate** | **Certainty** | **Indirect estimate** | **Certainty** | **Network estimate** | **Certainty** |
| --- | --- | --- | --- | --- | --- | --- |
| DPP4i vs Pla | 1.29 (0.11, 15.44) | High | 2.18 (0.18, 26.97) | Low^4^ | 1.67 (0.29, 9.78) | Moderate^7^ |
| SGLT2i vs DPP4i | 1.32 (0.19, 9.05) | Moderate1 | 0.68 (0.01,41.04) | Low^6^ | 1.17 (0.21, 6.69) | Low^7^ |
| SGLT2i vs GLP1RAs | 0.33 (0.01, 8.08) | High | - | - | 0.33 (0.01, 8.08) | Moderate^7^ |
| SGLT2i vs Met | 1.27 (0.11,15.25) | Moderate1 | - | - | 1.27 (0.11,15.25) | Low^7^ |
| SGLT2i vs Pla | 1.41 (0.89, 2.25) | Low1,2 | 27.76 (0.01, 130776.25) | Moderate^5^ | 1.43 (0.90, 2.27) | Low^7^ |
| SGLT2i vs SU | 0.97 (0.12, 7.92) | Moderate1 | - | - | 0.97 (0.12, 7.92) | Low^7^ |
| SGLT2i vs Tzds | 1.02 (0.06,16.57) | Moderate1 | - | - | 1.02 (0.06,16.57) | Low^7^ |
| DPP4i vs GLP1RAs | - | - | 0.38 (0.01,14.76) | Moderate^5^ | 0.38 (0.01,14.76) | Low^7^ |
| DPP4i vs Met | - | - | 1.50 (0.07,30.99) | Moderate^3^ | 1.50 (0.07,30.99) | Low^7^ |
| DPP4i vs SU | - | - | 1.14 (0.07,17.43) | Moderate^3^ | 1.14 (0.07,17.43) | Low^7^ |
| DPP4i vs Tzds | - | - | 1.19 (0.04,32.00) | Moderate^3^ | 1.19 (0.04,32.00) | Low^7^ |
| GLP1RAs vs Met | - | - | 3.89 (0.07, 224.28) | Moderate^5^ | 3.89 (0.07, 224.28) | Low^7^ |
| GLP1RAs vs Pla | - | - | 4.35 (0.17, 111.02) | Low^6^ | 4.35 (0.17, 111.02) | Very Low^7^ |
| GLP1RAs vs SU | - | - | 2.97 (0.06, 136.98) | Moderate^3^ | 2.97 (0.06, 136.98) | Low^7^ |
| GLP1RAs vs Tzds | - | - | 3.10 (0.04, 217.69) | Moderate^3^ | 3.10 (0.04, 217.69) | Low^7^ |
| Met vs Pla | - | - | 1.12 (0.09,13.96) | Low^4^ | 1.12 (0.09,13.96) | Very Low^7^ |
| Met vs SU | - | - | 0.76 (0.03,19.67) | Moderate^3^ | 0.76 (0.03,19.67) | Low^7^ |
| Met vs Tzds | - | - | 0.80 (0.02,33.40) | Moderate^3^ | 0.80 (0.02,33.40) | Low^7^ |
| Pla vs SU | - | - | 0.68 (0.08, 5.85) | Low^4^ | 0.68 (0.08, 5.85) | Very Low^7^ |
| Pla vs Tzds | - | - | 0.71 (0.04,12.08) | Low^4^ | 0.71 (0.04,12.08) | Very Low^7^ |
| SU vs Tzds | - | - | 1.04 (0.03,34.26) | Moderate^3^ | 1.04 (0.03,34.26) | Low^7^ |

**Notes:** 1, Risk of bias; 2, Publication bias; 3, Contributing direct evidence of moderate quality; 4, Contributing direct evidence of moderate or low quality; 5, Contributing direct evidence of high or moderate quality; 6, Contributing direct evidence of high or low quality; 7 Imprecision.

## Supplementary Table 8. GRADE assessments for different doses of SGLT-2i

| **Comparison** | **Direct estimate** | **Certainty** | **Indirect estimate** | **Certainty** | **Network estimate** | **Certainty** |
| --- | --- | --- | --- | --- | --- | --- |
| Active vs Cana 100mg | 1.94 (0.16, 23.24) | High | 0.25 (0.04, 1.62) | Moderate2 | 0.52 (0.12, 2.35) | Low^5^ |
| Active vs Cana 300mg | 1.00 (0.10, 9.67) | High | 0.49 (0.08, 3.06) | Moderate2 | 0.65 (0.16, 2.70) | Low^5^ |
| Active vs Dapa 10mg | 1.95 (0.16, 23.34) | Moderate^1^ | 0.47 (0.12, 1.92) | Moderate2 | 0.67 (0.20, 2.25) | Low^5^ |
| Active vs Dapa 5mg | 1.03 (0.02, 53.24) | Moderate^1^ | 2.63 (0.04, 157.95) | Moderate2 | 1.62 (0.09, 27.72) | Moderate^5^ |
| Active vs Empa 10mg | 0.40 (0.03, 4.73) | Moderate^1^ | 1.85 (0.33, 10.44) | Moderate3 | 1.11 (0.27, 4.61) | Moderate^5^ |
| Active vs Empa 25mg | 0.51 (0.06, 4.14) | Moderate^1^ | 3.07 (0.47, 20.18) | Moderate3 | 1.38 (0.34, 5.59) | Moderate^5^ |
| Active vs Ertu 15mg | 0.52 (0.04, 6.21) | High | 1.31 (0.13, 12.93) | Moderate2 | 0.86 (0.16, 4.60) | Moderate^5^ |
| Active vs Ertu 5mg | 1.02 (0.06, 16.32) | High | 1.29 (0.13, 12.76) | Moderate2 | 1.17 (0.20, 6.86) | Low^5^ |
| Active vs Ipra 50mg | 0.94 (0.02, 48.88) | High | 1.39 (0.02, 83.81) | Moderate2 | 1.14 (0.07, 19.53) | Low^5^ |
| Active vs Pla | 1.29 (0.11, 15.44) | High | 1.23 (0.35, 4.30) | Moderate3 | 1.24 (0.41, 3.80) | Moderate^5^ |
| Bexa 20mg vs Pla | 0.99 (0.02, 50.07) | Moderate^1^ | - | - | 0.99 (0.02, 50.07) | Moderate^5^ |
| Cana 100mg vs Cana 300mg | 0.74 (0.14, 3.92) | Moderate^1^ | 3.48 (0.33, 36.91) | Moderate2 | 1.24 (0.32, 4.85) | Low^5^ |
| Cana 100mg vs Pla | 3.29 (0.83, 13.01) | Moderate^1^ | 0.72 (0.05, 9.72) | High4 | 2.37 (0.70, 7.97) | Low^5^ |
| Cana 300mg vs Pla | 1.43 (0.38, 5.33) | Moderate^1^ | 4.97 (0.46, 53.67) | High4 | 1.91 (0.60, 6.06) | Low^5^ |
| Dapa 10mg vs Pla | 2.00 (1.05, 3.81) | Moderate^1^ | 0.49 (0.03, 7.81) | Moderate2 | 1.86 (1.00, 3.48) | Low^5^ |
| Dapa 5mg vs Pla | 0.49 (0.01, 24.99) | Moderate^1^ | 1.25 (0.02, 76.36) | Moderate2 | 0.77 (0.04, 13.15) | Low^5^ |
| Empa 10mg vs Empa 25mg | 1.33 (0.37, 4.76) | Moderate^1^ | 0.32 (0.00, 78.38) | Moderate3 | 1.24 (0.36, 4.28) | Low^5^ |
| Empa 10mg vs Pla | 0.95 (0.28, 3.20) | Moderate^1^ | 2.95 (0.15, 59.38) | Moderate3 | 1.12 (0.36, 3.43) | Low^5^ |
| Empa 25mg vs Pla | 0.70 (0.19, 2.59) | Moderate1 | 2.27 (0.18, 27.98) | Moderate3 | 0.90 (0.28, 2.88) | Low^5^ |
| Ertu 15mg vs Ertu 5mg | 1.31 (0.28, 6.10) | Moderate1 | 69.22 (0.00, 115449270.86) | Moderate3 | 1.37 (0.30, 6.33) | Low^5^ |
| Ertu 15mg vs Pla | 1.00 (0.14, 7.14) | Moderate1 | 2.90 (0.20, 42.83) | Moderate3 | 1.45 (0.30, 7.09) | Low^5^ |
| Ertu 5mg vs Pla | 0.99 (0.14, 7.04) | Moderate1 | 1.25 (0.06, 25.53) | Moderate3 | 1.06 (0.20, 5.49) | Low^5^ |
| Hena 10mg vs Hena 5mg | 1.01 (0.02, 51.34) | High | - | - | 1.01 (0.02, 51.34) | Low^5^ |
| Hena 10mg vs Pla | 1.01 (0.02, 51.02) | High | - | - | 1.01 (0.02, 51.02) | Low^5^ |
| Hena 5mg vs Pla | 0.99 (0.02, 50.39) | High | - | - | 0.99 (0.02, 50.39) | Low^5^ |
| Ipra 50mg vs Pla | 0.90 (0.02, 46.24) | Moderate1 | 1.34 (0.02, 81.68) | High4 | 1.09 (0.06, 18.72) | Low^5^ |
| Pla vs Tofo 20mg | 1.99 (0.04,101.49) | Moderate1 | - | - | 1.99 (0.04,101.49) | Low^5^ |
| Active vs Bexa 20mg | - | - | 1.26 (0.02, 74.56) | Moderate2 | 1.26 (0.02, 74.56) | Low^5^ |
| Active vs Hena 10mg | - | - | 1.23 (0.02, 73.15) | High4 | 1.23 (0.02, 73.15) | Low^5^ |
| Active vs Hena 5mg | - | - | 1.25 (0.02, 74.05) | High4 | 1.25 (0.02, 74.05) | Low^5^ |
| Active vs Tofo 20mg | - | - | 2.47 (0.04,147.29) | Moderate2 | 2.47 (0.04,147.29) | Moderate^5^ |
| Bexa 20mg vs Cana 100mg | - | - | 0.42 (0.01, 25.42) | Moderate3 | 0.42 (0.01, 25.42) | Moderate^5^ |
| Bexa 20mg vs Cana 300mg | - | - | 0.52 (0.01, 30.93) | Moderate3 | 0.52 (0.01, 30.93) | Low^5^ |
| Bexa 20mg vs Dapa 10mg | - | - | 0.53 (0.01, 28.21) | Moderate3 | 0.53 (0.01, 28.21) | Low^5^ |
| Bexa 20mg vs Dapa 5mg | - | - | 1.29 (0.01,163.62) | Moderate3 | 1.29 (0.01,163.62) | Low^5^ |
| Bexa 20mg vs Empa 10mg | - | - | 0.89 (0.01, 52.57) | Moderate3 | 0.89 (0.01, 52.57) | Low^5^ |
| Bexa 20mg vs Empa 25mg | - | - | 1.10 (0.02, 65.73) | Moderate3 | 1.10 (0.02, 65.73) | Low^5^ |
| Bexa 20mg vs Ertu 15mg | - | - | 0.68 (0.01, 46.95) | Moderate3 | 0.68 (0.01, 46.95) | Low^5^ |
| Bexa 20mg vs Ertu 5mg | - | - | 0.93 (0.01, 65.75) | Moderate3 | 0.93 (0.01, 65.75) | Low^5^ |
| Bexa 20mg vs Hena 10mg | - | - | 0.98 (0.00,252.99) | Moderate2 | 0.98 (0.00,252.99) | Low^5^ |
| Bexa 20mg vs Hena 5mg | - | - | 0.99 (0.00,256.14) | Moderate2 | 0.99 (0.00,256.14) | Moderate^5^ |
| Bexa 20mg vs Ipra 50mg | - | - | 0.90 (0.01,115.22) | Moderate3 | 0.90 (0.01,115.22) | Moderate^5^ |
| Bexa 20mg vs Tofo 20mg | - | - | 1.97 (0.01,508.89) | Moderate3 | 1.97 (0.01,508.89) | Low^5^ |
| Cana 100mg vs Dapa 10mg | - | - | 1.27 (0.33, 4.92) | Moderate2 | 1.27 (0.33, 4.92) | Low^5^ |
| Cana 100mg vs Dapa 5mg | - | - | 3.08 (0.15, 65.12) | Moderate2 | 3.08 (0.15, 65.12) | Low^5^ |
| Cana 100mg vs Empa 10mg | - | - | 2.12 (0.42, 10.79) | Moderate2 | 2.12 (0.42, 10.79) | Low^5^ |
| Cana 100mg vs Empa 25mg | - | - | 2.63 (0.51, 13.58) | Moderate2 | 2.63 (0.51, 13.58) | Low^5^ |
| Cana 100mg vs Ertu 15mg | - | - | 1.63 (0.23, 11.49) | Moderate2 | 1.63 (0.23, 11.49) | Low^5^ |
| Cana 100mg vs Ertu 5mg | - | - | 2.23 (0.30, 16.59) | Moderate2 | 2.23 (0.30, 16.59) | Low^5^ |
| Cana 100mg vs Hena 10mg | - | - | 2.35 (0.04,143.24) | High4 | 2.35 (0.04,143.24) | Low^5^ |
| Cana 100mg vs Hena 5mg | - | - | 2.38 (0.04,145.02) | High4 | 2.38 (0.04,145.02) | Low^5^ |
| Cana 100mg vs Ipra 50mg | - | - | 2.17 (0.10, 45.88) | Moderate2 | 2.17 (0.10, 45.88) | Low^5^ |
| Cana 100mg vs Tofo 20mg | - | - | 4.72 (0.08,288.43) | Moderate2 | 4.72 (0.08,288.43) | Low^5^ |
| Cana 300mg vs Dapa 10mg | - | - | 1.03 (0.28, 3.76) | Moderate2 | 1.03 (0.28, 3.76) | Low^5^ |
| Cana 300mg vs Dapa 5mg | - | - | 2.49 (0.12, 51.09) | Moderate2 | 2.49 (0.12, 51.09) | Low^5^ |
| Cana 300mg vs Empa 10mg | - | - | 1.71 (0.35, 8.30) | Moderate2 | 1.71 (0.35, 8.30) | Low^5^ |
| Cana 300mg vs Empa 25mg | - | - | 2.12 (0.43, 10.44) | Moderate2 | 2.12 (0.43, 10.44) | Low^5^ |
| Cana 300mg vs Ertu 15mg | - | - | 1.32 (0.20, 8.88) | Moderate2 | 1.32 (0.20, 8.88) | Low^5^ |
| Cana 300mg vs Ertu 5mg | - | - | 1.80 (0.25, 12.85) | Moderate2 | 1.80 (0.25, 12.85) | Low^5^ |
| Cana 300mg vs Hena 10mg | - | - | 1.90 (0.03,113.74) | High4 | 1.90 (0.03,113.74) | Low^5^ |
| Cana 300mg vs Hena 5mg | - | - | 1.92 (0.03,115.16) | High4 | 1.92 (0.03,115.16) | Low^5^ |
| Cana 300mg vs Ipra 50mg | - | - | 1.75 (0.09, 35.99) | Moderate2 | 1.75 (0.09, 35.99) | Low^5^ |
| Cana 300mg vs Tofo 20mg | - | - | 3.81 (0.06,229.05) | Moderate2 | 3.81 (0.06,229.05) | Low^5^ |
| Dapa 10mg vs Dapa 5mg | - | - | 2.43 (0.13, 43.93) | Moderate3 | 2.43 (0.13, 43.93) | Low^5^ |
| Dapa 10mg vs Empa 10mg | - | - | 2.43 (0.13, 43.93) | Moderate3 | 2.43 (0.13, 43.93) | Low^5^ |
| Dapa 10mg vs Empa 25mg | - | - | 2.07 (0.56, 7.62) | Moderate3 | 2.07 (0.56, 7.62) | Low^5^ |
| Dapa 10mg vs Ertu 15mg | - | - | 1.28 (0.24, 6.94) | Moderate3 | 1.28 (0.24, 6.94) | Low^5^ |
| Dapa 10mg vs Ertu 5mg | - | - | 1.76 (0.31, 10.07) | Moderate2 | 1.76 (0.31, 10.07) | Low^5^ |
| Dapa 10mg vs Hena 10mg | - | - | 1.85 (0.03, 98.73) | Moderate2 | 1.85 (0.03, 98.73) | Low^5^ |
| Dapa 10mg vs Hena 5mg | - | - | 1.88 (0.04, 99.96) | Moderate2 | 1.88 (0.04, 99.96) | Low^5^ |
| Dapa 10mg vs Ipra 50mg | - | - | 1.71 (0.09, 30.95) | Moderate2 | 1.71 (0.09, 30.95) | Low^5^ |
| Dapa 10mg vs Tofo 20mg | - | - | 3.72 (0.07,198.84) | Moderate3 | 3.72 (0.07,198.84) | Low^5^ |
| Dapa 5mg vs Empa 10mg | - | - | 0.69 (0.03, 14.03) | Moderate3 | 0.69 (0.03, 14.03) | Low^5^ |
| Dapa 5mg vs Empa 25mg | - | - | 0.85 (0.04, 17.40) | Moderate3 | 0.85 (0.04, 17.40) | Low^5^ |
| Dapa 5mg vs Ertu 15mg | - | - | 0.53 (0.02, 12.74) | Moderate2 | 0.53 (0.02, 12.74) | Low^5^ |
| Dapa 5mg vs Ertu 5mg | - | - | 0.72 (0.03, 18.11) | Moderate2 | 0.72 (0.03, 18.11) | Low^5^ |
| Dapa 5mg vs Hena 10mg | - | - | 0.76 (0.01, 97.09) | Moderate2 | 0.76 (0.01, 97.09) | Low^5^ |
| Dapa 5mg vs Hena 5mg | - | - | 0.77 (0.01, 98.29) | Moderate2 | 0.77 (0.01, 98.29) | Low^5^ |
| Dapa 5mg vs Ipra 50mg | - | - | 0.70 (0.01, 36.20) | Moderate2 | 0.70 (0.01, 36.20) | Low^5^ |
| Dapa 5mg vs Tofo 20mg | - | - | 1.53 (0.01,195.38) | Moderate3 | 1.53 (0.01,195.38) | Low^5^ |
| Empa 10mg vs Ertu 15mg | - | - | 0.77 (0.12, 5.11) | Moderate2 | 0.77 (0.12, 5.11) | Low^5^ |
| Empa 10mg vs Ertu 5mg | - | - | 1.05 (0.15, 7.40) | Moderate2 | 1.05 (0.15, 7.40) | Low^5^ |
| Empa 10mg vs Hena 10mg | - | - | 1.11 (0.02, 65.79) | Moderate2 | 1.11 (0.02, 65.79) | Low^5^ |
| Empa 10mg vs Hena 5mg | - | - | 1.12 (0.02, 66.61) | Moderate2 | 1.12 (0.02, 66.61) | Low^5^ |
| Empa 10mg vs Ipra 50mg | - | - | 1.02 (0.05, 20.85) | Moderate2 | 1.02 (0.05, 20.85) | Low^5^ |
| Empa 10mg vs Tofo 20mg | - | - | 2.22 (0.04,132.48) | Moderate3 | 2.22 (0.04,132.48) | Low^5^ |
| Empa 25mg vs Ertu 15mg | - | - | 0.62 (0.09, 4.17) | Moderate2 | 0.62 (0.09, 4.17) | Low^5^ |
| Empa 25mg vs Ertu 5mg | - | - | 0.85 (0.12, 6.03) | Moderate2 | 0.85 (0.12, 6.03) | Low^5^ |
| Empa 25mg vs Hena 10mg | - | - | 0.90 (0.01, 53.70) | Moderate2 | 0.90 (0.01, 53.70) | Low^5^ |
| Empa 25mg vs Hena 5mg | - | - | 0.91 (0.02, 54.37) | Moderate2 | 0.91 (0.02, 54.37) | Moderate^5^ |
| Empa 25mg vs Ipra 50mg | - | - | 0.83 (0.04, 16.88) | Moderate2 | 0.83 (0.04, 16.88) | Moderate^5^ |
| Empa 25mg vs Tofo 20mg | - | - | 1.80 (0.03,108.14) | Moderate3 | 1.80 (0.03,108.14) | Moderate^5^ |
| Ertu 15mg vs Hena 10mg | - | - | 1.44 (0.02, 99.54) | Moderate2 | 1.44 (0.02, 99.54) | Moderate^5^ |
| Ertu 15mg vs Hena 5mg | - | - | 1.46 (0.02,100.78) | Moderate2 | 1.46 (0.02,100.78) | Low^5^ |
| Ertu 15mg vs Ipra 50mg | - | - | 1.33 (0.06, 32.06) | Moderate2 | 1.33 (0.06, 32.06) | Low^5^ |
| Ertu 15mg vs Tofo 20mg | - | - | 2.89 (0.04,200.41) | Moderate3 | 2.89 (0.04,200.41) | Moderate^5^ |
| Ertu 5mg vs Hena 10mg | - | - | 1.05 (0.01, 74.35) | Moderate2 | 1.05 (0.01, 74.35) | Moderate^5^ |
| Ertu 5mg vs Hena 5mg | - | - | 1.07 (0.02, 75.28) | Moderate2 | 1.07 (0.02, 75.28) | Low^5^ |
| Ertu 5mg vs Ipra 50mg | - | - | 0.97 (0.04, 24.31) | Moderate2 | 0.97 (0.04, 24.31) | Low^5^ |
| Ertu 5mg vs Tofo 20mg | - | - | 2.11 (0.03,149.70) | Moderate3 | 2.11 (0.03,149.70) | Low^5^ |
| Hena 10mg vs Ipra 50mg | - | - | 0.92 (0.01,117.41) | Moderate2 | 0.92 (0.01,117.41) | Low^5^ |
| Hena 10mg vs Tofo 20mg | - | - | 2.01 (0.01,518.58) | Moderate2 | 2.01 (0.01,518.58) | Low^5^ |
| Hena 5mg vs Ipra 50mg | - | - | 0.91 (0.01,115.96) | Moderate2 | 0.91 (0.01,115.96) | Low^5^ |
| Hena 5mg vs Tofo 20mg | - | - | 1.98 (0.01,512.18) | Moderate2 | 1.98 (0.01,512.18) | Low^5^ |
| Ipra 50mg vs Tofo 20mg | - | - | 2.17 (0.02,277.90) | Moderate3 | 2.17 (0.02,277.90) | Low^5^ |

**Footnote:**

1, Risk of bias; 2, Contributing direct evidence of high or moderate quality; 3, Contributing direct evidence of moderate quality; 4, Contributing direct evidence of high quality; 5. Imprecision.

SGLT-2i: Sodium-glucose co-transporter-2 inhibitors, Cana: Canagliflozin, Dapa: Dapagliflozin, Ertu: Ertugliflozin, Ipra: Ipragliflozin, Hena: Henagliflozin, Bexa: Bexagliflozin, Empa: Empagliflozin, Tofo: Tofogliflozin, Pla: Placebo, DKA: diabetic ketoacidosis.

##

## Supplementary Table 9. Frequentist P-score for different kinds of active antidiabetic drugs

| **The risk of DKA (odds ratio; 95% confidence interval)** | | |
| --- | --- | --- |
| **Intervention** | **Intervention vs Pla** | **Frequentist P-score** |
| GLP-1RAs | 4.35 (0.17, 111.02) | 0.7361 |
| DPP4i | 1.67 (0.29, 9.78) | 0.5456 |
| SGLT2i | 1.43 (0.90, 2.27) | 0.5298 |
| SU | 1.46 (0.17, 12.53) | 0.4951 |
| Tzds | 1.40 (0.08, 23.77) | 0.4808 |
| Met | 1.12 (0.09, 13.96) | 0.4166 |
| Pla | - | 0.2959 |

**Footnote:** GLP-1RAs: The glucagon-like peptide-1 receptor agonists; DPP4i: Dipeptidyl peptidase-4 inhibitor; SGLT2i: Sodium-glucose cotransporter-2 inhibitors; SU: Sulphonylurea; Tzds: Thiazolidinediones; Met: Metformin; Pla: Placebo; DKA: Diabetic ketoacidosis.

## Supplementary Table 10. Frequentist P-score for different doses of SGLT2i

| **The risk of DKA (odds ratio; 95% confidence interval)** | | |
| --- | --- | --- |
| **Intervention** | **Intervention vs Pla** | **Frequentist P-score** |
| Cana 100mg | 2.37 (0.70, 7.97) | 0.7388 |
| Dapa 10mg | 1.86 (1.00, 3.48) | 0.6815 |
| Cana 300mg | 1.91 (0.60, 6.06) | 0.6685 |
| Ertu 15mg | 1.45 (0.30, 7.09) | 0.5647 |
| Ipra 50mg | 1.09 (0.06,18.72) | 0.4809 |
| Hena 10mg | 1.01 (0.02,51.02) | 0.4728 |
| Hena 5mg | 0.99 (0.02,50.39) | 0.4705 |
| Bexa 20mg | 0.99 (0.02,50.07) | 0.4693 |
| Empa 10mg | 1.12 (0.36, 3.43) | 0.4661 |
| Ertu 5mg | 1.06 (0.20, 5.49) | 0.4531 |
| Dapa 5mg | 0.77 (0.04,13.15) | 0.3992 |
| Empa 25mg | 0.90 (0.28, 2.88) | 0.3826 |
| Tofo 20mg | 0.50 (0.01,25.55) | 0.3491 |

**Footnote:** Cana: Canagliflozin; Dapa: Dapagliflozin; Ertu: Ertugliflozin; Ipra: Ipragliflozin; Hena: Henagliflozin; Bexa: Bexagliflozin; Empa: Empagliflozin; Tofo: Tofogliflozin; Pla: Placebo; DKA: Diabetic ketoacidosis

## Supplementary Table 11. Results of sensitivity analyses for different kinds of active antidiabetic drugs

### **11.1 Exclusion of studies with fewer than 100 participants**

Outcome: The risk of DKA (odds ratio; 95% confidence interval)

| **DPP4i** |  |  |  |  |  |
| --- | --- | --- | --- | --- | --- |
| 0.38 (0.01, 14.76) | **GLP1RAs** |  |  |  |  |
| 1.50 (0.07, 30.99) | 3.89 (0.07,224.28) | **Met** |  |  |  |
| 1.67 (0.29, 9.78) | 4.35 (0.17,111.02) | 1.12 (0.09, 13.96) | **Pla** |  |  |
| 1.17 (0.21, 6.69) | 3.05 (0.12, 75.34) | 0.78 (0.07, 9.38) | 0.70 (0.44, 1.12) | **SGLT2i** |  |
| 1.14 (0.07, 17.43) | 2.97 (0.06,136.98) | 0.76 (0.03, 19.67) | 0.68 (0.08, 5.85) | 0.97 (0.12, 7.92) | **SU** |

**Footnote:** DPP4i: Dipeptidyl peptidase-4 inhibitor; GLP-1RAs: The glucagon-like peptide-1 receptor agonists; SGLT2i: Sodium-glucose cotransporter-2 inhibitors; SU: Sulphonylurea; Met: Metformin; Pla: Placebo; DKA: Diabetic ketoacidosis.

### **11.2 Exclusion of studies with treatment duration <24 weeks**

Outcome: The risk of DKA (odds ratio; 95% confidence interval)

| **DPP4i** |  |  |  |  |  |  |
| --- | --- | --- | --- | --- | --- | --- |
| 0.38 (0.01, 14.49) | **GLP1RAs** |  |  |  |  |  |
| 1.47 (0.07, 30.44) | 3.89 (0.07,224.28) | **Met** |  |  |  |  |
| 1.75 (0.30, 10.24) | 4.63 (0.18,118.34) | 1.19 (0.10, 14.89) | **Pla** |  |  |  |
| 1.15 (0.20, 6.57) | 3.05 (0.12, 75.34) | 0.78 (0.07, 9.38) | 0.66 (0.41, 1.06) | **SGLT2i** |  |  |
| 1.12 (0.07, 17.12) | 2.97 (0.06,136.98) | 0.76 (0.03, 19.67) | 0.64 (0.07, 5.50) | 0.97 (0.12, 7.92) | **SU** |  |
| 1.17 (0.04, 31.43) | 3.10 (0.04,217.69) | 0.80 (0.02, 33.40) | 0.67 (0.04, 11.37) | 1.02 (0.06, 16.57) | 1.04 (0.03, 34.26) | **Tzds** |

**Footnote:** DPP4i: Dipeptidyl peptidase-4 inhibitor; SGLT2i: Sodium-glucose cotransporter-2 inhibitors; GLP-1RAs: The glucagon-like peptide-1 receptor agonists; SU: Sulphonylurea; Tzds: Thiazolidinediones; Met: Metformin; Pla: Placebo; DKA: Diabetic ketoacidosis.

### **11.3 Exclusion of studies without a placebo-control**

Outcome: The risk of DKA (odds ratio; 95% confidence interval)

| **DPP4i** |  |  |  |
| --- | --- | --- | --- |
| 1.67 (0.29， 9.78) | **Pla** |  |  |
| 1.17 (0.21， 6.69) | 0.70 (0.44， 1.12) | **SGLT2i** |  |
| 1.14 (0.02，85.28) | 0.68 (0.01，36.32) | 0.97 (0.02，50.36) | **Tzds** |

**Footnote:** DPP4i: Dipeptidyl peptidase-4 inhibitor; SGLT2i: Sodium-glucose cotransporter-2 inhibitors; Tzds: Thiazolidinediones; Pla: Placebo; DKA: Diabetic ketoacidosis.

### **11.4 Exclusion of the risk of bias arising from the randomization process was ‘Some concerns’**

Outcome: The risk of DKA (odds ratio; 95% confidence interval)

| **DPP4i** |  |  |  |  |  |  |
| --- | --- | --- | --- | --- | --- | --- |
| 0.42 (0.01, 16.24) | **GLP1RAs** |  |  |  |  |  |
| 1.64 (0.08, 34.13) | 3.89 (0.07,224.28) | **Met** |  |  |  |  |
| 1.33 (0.22, 8.07) | 3.16 (0.12, 83.76) | 0.81 (0.06, 10.65) | **Pla** |  |  |  |
| 1.29 (0.22, 7.39) | 3.05 (0.12, 75.34) | 0.78 (0.07, 9.38) | 0.97 (0.49, 1.91) | **SGLT2i** |  |  |
| 1.25 (0.08, 19.20) | 2.97 (0.06,136.98) | 0.76 (0.03, 19.67) | 0.94 (0.10, 8.53) | 0.97 (0.12, 7.92) | **SU** |  |
| 1.37 (0.02,102.59) | 3.24 (0.02,524.46) | 0.83 (0.01, 88.34) | 1.03 (0.02, 56.45) | 1.06 (0.02, 55.08) | 1.09 (0.01, 95.35) | **Tzds** |

**Footnote:** DPP4i: Dipeptidyl peptidase-4 inhibitor; GLP-1RAs: The glucagon-like peptide-1 receptor agonists; SGLT2i: Sodium-glucose cotransporter-2 inhibitors; SU: Sulphonylurea; Tzds: Thiazolidinediones; Met: Metformin; Pla: Placebo; DKA: Diabetic ketoacidosis.

### **11.5 Exclusion of studies that the risk of DKA was 0 percent**

Outcome: The risk of DKA (odds ratio; 95% confidence interval)

| **DPP4i** |  |  |  |  |  |
| --- | --- | --- | --- | --- | --- |
| 0.37 (0.01, 15.64) | **GLP1RAs** |  |  |  |  |
| 1.69 (0.04, 71.60) | 4.58 (0.05,426.40) | **Met** |  |  |  |
| 2.02 (0.28, 14.36) | 5.48 (0.21,140.99) | 1.19 (0.05, 30.71) | **Pla** |  |  |
| 1.12 (0.16, 7.85) | 3.05 (0.12, 75.34) | 0.67 (0.03, 16.41) | 0.56 (0.33, 0.94) | **SGLT2i** |  |
| 1.66 (0.04, 70.42) | 4.51 (0.05,419.43) | 0.98 (0.01, 91.40) | 0.82 (0.03, 21.16) | 1.48 (0.06, 36.38) | **SU** |

**Footnote:** DPP4i: Dipeptidyl peptidase-4 inhibitor; GLP-1RAs: The glucagon-like peptide-1 receptor agonists; SGLT2i: Sodium-glucose cotransporter-2 inhibitors; SU: Sulphonylurea; Met: Metformin; Pla: Placebo; DKA: Diabetic ketoacidosis.

### **11.6 This analysis was estimated in a Bayesian framework**

Outcome: The risk of DKA (odds ratio; 95% confidence interval)

| **DPP4i** |  |  |  |  |  |
| --- | --- | --- | --- | --- | --- |
| 0 (0, 1.89) | **GLP1RAs** |  |  |  |  |
| 488210.35 (0.08, 18574362750430658560) | 36081335297217802240 (443.37, 9.21887616170278e+47) | **Met** |  |  |  |
| 4.77 (0.04, 592.73) | 10248674968210.4 (4.2, 8.9426292454194e+40) | 0 (0, 25.65) | **Pla** |  |  |
| 1.72 (0.02, 178.32) | 3585480113363.9 (1.64, 3.2494506701051e+40) | 0 (0, 8.13) | 0.36 (0.07, 1.88) | **SGLT2i** |  |
| 17489500511763.2 (0.73, 4.59992874104026e+46) | 1.54719695613833e+28 (199270.64, 1.93933747055289e+67) | 12085165.07 (0, 3.60389018552562e+41) | 3573764810931.76 (0.28, 4.71022789799015e+45) | 10190588686693.9 (0.88, 1.41417682286124e+46) | **Su** |
| 3930953011.97 (0, 3.54150321477861e+39) | 7.62109008974112e+23 (0, 1.65379875916536e+63) | 1521.62 (0, 9.16087062794269e+33) | 774629991.04 (0, 5.75082238306522e+38) | 2170966452.84 (0, 1.65224795256838e+39) | 0 (0, 7.32328375838767e+30) |

**Footnote:** DPP4i: Dipeptidyl peptidase-4 inhibitor; GLP-1RAs: The glucagon-like peptide-1 receptor agonists; SGLT2i: Sodium-glucose cotransporter-2 inhibitors; SU: Sulphonylurea; Met: Metformin; Pla: Placebo; DKA: Diabetic ketoacidosis.

## Supplementary Table 12. Results of sensitivity analyses for different doses of SGLT-2i

### **12.1 Exclusion of studies with fewer than 100 participants**

Outcome: The risk of DKA (odds ratio; 95% confidence interval)

| **Active** |  |  |  |  |  |  |  |  |  |  |  |  |  |  |
| --- | --- | --- | --- | --- | --- | --- | --- | --- | --- | --- | --- | --- | --- | --- |
| 1.33 (0.02, 79.92) | **Bexa 20mg** |  |  |  |  |  |  |  |  |  |  |  |  |  |
| 0.55 (0.12, 2.52) | 0.41 (0.01, 25.16) | **Cana 100mg** |  |  |  |  |  |  |  |  |  |  |  |  |
| 0.68 (0.16, 2.89) | 0.51 (0.01, 30.57) | 1.24 (0.32, 4.84) | **Cana 300mg** |  |  |  |  |  |  |  |  |  |  |  |
| 0.70 (0.20, 2.47) | 0.53 (0.01, 28.11) | 1.28 (0.33, 4.96) | 1.03 (0.28, 3.80) | **Dapa 10mg** |  |  |  |  |  |  |  |  |  |  |
| 2.68 (0.04,161.97) | 2.02 (0.01,521.85) | 4.88 (0.08,299.21) | 3.95 (0.07,238.04) | 3.82 (0.07,204.73) | **Dapa 5mg** |  |  |  |  |  |  |  |  |  |
| 1.16 (0.27, 4.94) | 0.88 (0.01, 52.02) | 2.12 (0.42, 10.78) | 1.72 (0.35, 8.31) | 1.66 (0.46, 5.94) | 0.43 (0.01, 25.96) | **Empa 10mg** |  |  |  |  |  |  |  |  |
| 1.44 (0.35, 5.96) | 1.08 (0.02, 64.86) | 2.62 (0.51, 13.53) | 2.12 (0.43, 10.42) | 2.05 (0.56, 7.55) | 0.54 (0.01, 32.36) | 1.23 (0.36, 4.27) | **Empa 25mg** |  |  |  |  |  |  |  |
| 0.89 (0.16, 4.83) | 0.67 (0.01, 46.06) | 1.61 (0.23, 11.38) | 1.31 (0.19, 8.81) | 1.26 (0.23, 6.85) | 0.33 (0.00, 22.98) | 0.76 (0.11, 5.07) | 0.62 (0.09, 4.14) | **Ertu 15mg** |  |  |  |  |  |  |
| 1.22 (0.20, 7.22) | 0.91 (0.01, 64.63) | 2.21 (0.30, 16.46) | 1.79 (0.25, 12.77) | 1.73 (0.30, 9.94) | 0.45 (0.01, 32.24) | 1.04 (0.15, 7.35) | 0.85 (0.12, 6.01) | 1.37 (0.30, 6.34) | **Ertu 5mg** |  |  |  |  |  |
| 1.31 (0.02, 78.40) | 0.98 (0.00,252.99) | 2.38 (0.04,144.84) | 1.92 (0.03,115.23) | 1.86 (0.03, 99.09) | 0.49 (0.00,126.04) | 1.12 (0.02, 66.55) | 0.91 (0.02, 54.52) | 1.47 (0.02,101.82) | 1.07 (0.02, 75.86) | **Hena 10mg** |  |  |  |  |
| 1.32 (0.02, 79.38) | 0.99 (0.00,256.14) | 2.41 (0.04,146.64) | 1.95 (0.03,116.66) | 1.88 (0.04,100.32) | 0.49 (0.00,127.61) | 1.13 (0.02, 67.38) | 0.92 (0.02, 55.20) | 1.49 (0.02,103.09) | 1.09 (0.02, 76.81) | 1.01 (0.02, 51.34) | **Hena 5mg** |  |  |  |
| 1.45 (0.02, 87.86) | 1.09 (0.00,282.95) | 2.64 (0.04,162.31) | 2.14 (0.04,129.13) | 2.07 (0.04,111.07) | 0.54 (0.00,140.97) | 1.25 (0.02, 74.58) | 1.01 (0.02, 61.10) | 1.64 (0.02,114.08) | 1.19 (0.02, 84.99) | 1.11 (0.00,288.33) | 1.10 (0.00,284.78) | **Ipra 50mg** |  |  |
| 1.31 (0.41, 4.22) | 0.99 (0.02, 50.07) | 2.39 (0.71, 8.07) | 1.94 (0.61, 6.15) | 1.87 (1.00, 3.50) | 0.49 (0.01, 24.99) | 1.13 (0.37, 3.48) | 0.91 (0.29, 2.93) | 1.48 (0.30, 7.27) | 1.08 (0.21, 5.62) | 1.01 (0.02, 51.02) | 0.99 (0.02, 50.39) | 0.90 (0.02, 46.24) | **Pla** |  |
| 2.62 (0.04,157.88) | 1.97 (0.01,508.89) | 4.77 (0.08,291.66) | 3.86 (0.06,232.03) | 3.73 (0.07,199.55) | 0.98 (0.00,253.54) | 2.25 (0.04,134.02) | 1.82 (0.03,109.79) | 2.95 (0.04,205.01) | 2.15 (0.03,152.74) | 2.01 (0.01,518.58) | 1.98 (0.01,512.18) | 1.80 (0.01,468.91) | 1.99 (0.04,101.49) | **Tofo 20mg** |

**Footnote:** SGLT-2i: Sodium-glucose co-transporter-2 inhibitors, Cana: Canagliflozin, Dapa: Dapagliflozin, Ertu: Ertugliflozin, Ipra: Ipragliflozin, Hena: Henagliflozin, Bexa: Bexagliflozin, Empa: Empagliflozin, Tofo: Tofogliflozin, Pla: Placebo, DKA: diabetic ketoacidosis.

### **12.2 Exclusion of studies with treatment duration <24 weeks**

Outcome: The risk of DKA (odds ratio; 95% confidence interval)

| **Active** |  |  |  |  |  |  |  |  |  |  |  |  |  |  |
| --- | --- | --- | --- | --- | --- | --- | --- | --- | --- | --- | --- | --- | --- | --- |
| 1.39 (0.02, 82.85) | **Bexa 20mg** |  |  |  |  |  |  |  |  |  |  |  |  |  |
| 0.57 (0.12, 2.58) | 0.41 (0.01, 24.96) | **Cana 100mg** |  |  |  |  |  |  |  |  |  |  |  |  |
| 0.70 (0.17, 2.96) | 0.51 (0.01, 30.29) | 1.23 (0.31, 4.84) | **Cana 300mg** |  |  |  |  |  |  |  |  |  |  |  |
| 0.73 (0.21, 2.53) | 0.53 (0.01, 28.04) | 1.28 (0.33, 4.98) | 1.04 (0.28, 3.82) | **Dapa 10mg** |  |  |  |  |  |  |  |  |  |  |
| 1.03 (0.02, 53.24) | 0.74 (0.00,218.31) | 1.81 (0.03,124.18) | 1.47 (0.02, 98.03) | 1.41 (0.02, 88.38) | **Dapa 5mg** |  |  |  |  |  |  |  |  |  |
| 1.00 (0.23, 4.37) | 0.72 (0.01, 43.92) | 1.76 (0.33, 9.44) | 1.43 (0.28, 7.29) | 1.37 (0.36, 5.26) | 0.97 (0.01, 65.80) | **Empa 10mg** |  |  |  |  |  |  |  |  |
| 1.25 (0.29, 5.36) | 0.91 (0.01, 55.73) | 2.21 (0.40, 12.10) | 1.79 (0.34, 9.33) | 1.72 (0.43, 6.83) | 1.22 (0.02, 81.82) | 1.25 (0.34, 4.63) | **Empa 25mg** |  |  |  |  |  |  |  |
| 0.91 (0.17, 4.93) | 0.66 (0.01, 45.33) | 1.60 (0.23, 11.29) | 1.30 (0.19, 8.75) | 1.25 (0.23, 6.75) | 0.88 (0.01, 64.79) | 0.91 (0.13, 6.29) | 0.72 (0.10, 5.09) | **Ertu 15mg** |  |  |  |  |  |  |
| 1.25 (0.21, 7.38) | 0.90 (0.01, 63.71) | 2.20 (0.30, 16.36) | 1.78 (0.25, 12.71) | 1.71 (0.30, 9.83) | 1.22 (0.02, 92.13) | 1.25 (0.17, 9.13) | 1.00 (0.13, 7.39) | 1.37 (0.30, 6.35) | **Ertu 5mg** |  |  |  |  |  |
| 1.36 (0.02, 81.28) | 0.98 (0.00,252.99) | 2.40 (0.04,145.95) | 1.94 (0.03,116.24) | 1.86 (0.03, 99.34) | 1.32 (0.00,389.14) | 1.36 (0.02, 82.44) | 1.08 (0.02, 66.73) | 1.50 (0.02,103.32) | 1.09 (0.02, 76.87) | **Hena 10mg** |  |  |  |  |
| 1.38 (0.02, 82.29) | 0.99 (0.00,256.14) | 2.43 (0.04,147.76) | 1.96 (0.03,117.69) | 1.89 (0.04,100.57) | 1.34 (0.00,393.98) | 1.37 (0.02, 83.46) | 1.10 (0.02, 67.56) | 1.51 (0.02,104.61) | 1.10 (0.02, 77.83) | 1.01 (0.02, 51.34) | **Hena 5mg** |  |  |  |
| 1.19 (0.07, 20.57) | 0.86 (0.01,109.97) | 2.10 (0.10, 44.58) | 1.71 (0.08, 35.06) | 1.64 (0.09, 29.74) | 1.16 (0.01,150.84) | 1.19 (0.06, 24.94) | 0.95 (0.05, 20.00) | 1.31 (0.05, 31.68) | 0.96 (0.04, 23.93) | 0.88 (0.01,112.07) | 0.87 (0.01,110.68) | **Ipra 50mg** |  |  |
| 1.37 (0.43, 4.32) | 0.99 (0.02, 50.07) | 2.41 (0.72, 8.12) | 1.95 (0.61, 6.20) | 1.88 (1.00, 3.51) | 1.33 (0.02, 81.17) | 1.37 (0.41, 4.55) | 1.09 (0.31, 3.80) | 1.50 (0.31, 7.37) | 1.09 (0.21, 5.69) | 1.01 (0.02, 51.02) | 0.99 (0.02, 50.39) | 1.15 (0.07, 19.70) | **Pla** |  |
| 2.73 (0.05,163.68) | 1.97 (0.01,508.89) | 4.80 (0.08,293.88) | 3.89 (0.06,234.08) | 3.74 (0.07,200.06) | 2.65 (0.01,782.70) | 2.72 (0.04,166.00) | 2.17 (0.04,134.37) | 3.00 (0.04,208.03) | 2.18 (0.03,154.77) | 2.01 (0.01,518.58) | 1.98 (0.01,512.18) | 2.28 (0.02,292.18) | 1.99 (0.04,101.49) | **Tofo 20mg** |

**Footnote:** SGLT-2i: Sodium-glucose co-transporter-2 inhibitors; Cana: Canagliflozin; Dapa: Dapagliflozin; Ertu: Ertugliflozin; Ipra: Ipragliflozin; Hena: Henagliflozin; Bexa: Bexagliflozin; Empa: Empagliflozin; Tofo: Tofogliflozin; Pla: Placebo; DKA: diabetic ketoacidosis.

### **12.3 Exclusion of studies without a placebo-control**

Outcome: The risk of DKA (odds ratio; 95% confidence interval)

| **Active** |  |  |  |  |  |  |  |  |  |  |  |  |  |  |
| --- | --- | --- | --- | --- | --- | --- | --- | --- | --- | --- | --- | --- | --- | --- |
| 1.40 (0.02, 93.51) | **Bexa 20mg** |  |  |  |  |  |  |  |  |  |  |  |  |  |
| 0.51 (0.08, 3.10) | 0.36 (0.01, 22.46) | **Cana 100mg** |  |  |  |  |  |  |  |  |  |  |  |  |
| 0.86 (0.14, 5.14) | 0.61 (0.01, 37.56) | 1.69 (0.37, 7.72) | **Cana 300mg** |  |  |  |  |  |  |  |  |  |  |  |
| 0.70 (0.14, 3.40) | 0.50 (0.01, 26.55) | 1.38 (0.33, 5.70) | 0.81 (0.20, 3.25) | **Dapa 10mg** |  |  |  |  |  |  |  |  |  |  |
| 1.71 (0.10, 30.57) | 1.22 (0.01,158.76) | 3.37 (0.15, 75.28) | 1.99 (0.09, 43.85) | 2.45 (0.13, 46.37) | **Dapa 5mg** |  |  |  |  |  |  |  |  |  |
| 1.32 (0.23, 7.72) | 0.94 (0.02, 56.60) | 2.60 (0.47, 14.44) | 1.54 (0.29, 8.29) | 1.89 (0.50, 7.12) | 0.77 (0.04, 16.71) | **Empa 10mg** |  |  |  |  |  |  |  |  |
| 1.73 (0.28, 10.50) | 1.23 (0.02, 75.72) | 3.41 (0.58, 19.89) | 2.01 (0.35, 11.43) | 2.48 (0.62, 9.96) | 1.01 (0.05, 22.44) | 1.31 (0.35, 4.93) | **Empa 25mg** |  |  |  |  |  |  |  |
| 1.30 (0.16, 10.81) | 0.92 (0.01, 68.78) | 2.55 (0.29, 22.25) | 1.51 (0.18, 12.85) | 1.86 (0.28, 12.21) | 0.76 (0.03, 21.02) | 0.98 (0.12, 8.06) | 0.75 (0.09, 6.42) | **Ertu 15mg** |  |  |  |  |  |  |
| 1.31 (0.16, 10.96) | 0.94 (0.01, 69.74) | 2.59 (0.30, 22.55) | 1.53 (0.18, 13.03) | 1.88 (0.29, 12.38) | 0.77 (0.03, 21.31) | 0.99 (0.12, 8.17) | 0.76 (0.09, 6.51) | 1.01 (0.18, 5.87) | **Ertu 5mg** |  |  |  |  |  |
| 1.38 (0.02, 91.74) | 0.98 (0.00,252.99) | 2.71 (0.04,168.46) | 1.60 (0.03, 98.31) | 1.97 (0.04,105.23) | 0.81 (0.01,105.11) | 1.04 (0.02, 62.60) | 0.80 (0.01, 48.96) | 1.06 (0.01, 79.20) | 1.05 (0.01, 78.12) | **Hena 10mg** |  |  |  |  |
| 1.39 (0.02, 92.88) | 0.99 (0.00,256.14) | 2.75 (0.04,170.56) | 1.62 (0.03, 99.53) | 2.00 (0.04,106.54) | 0.82 (0.01,106.42) | 1.05 (0.02, 63.38) | 0.81 (0.01, 49.57) | 1.08 (0.01, 80.19) | 1.06 (0.01, 79.09) | 1.01 (0.02, 51.34) | **Hena 5mg** |  |  |  |
| 1.53 (0.02,102.79) | 1.09 (0.00,282.95) | 3.02 (0.05,188.77) | 1.78 (0.03,110.16) | 2.19 (0.04,117.95) | 0.90 (0.01,117.65) | 1.16 (0.02, 70.15) | 0.89 (0.01, 54.86) | 1.18 (0.02, 88.73) | 1.17 (0.02, 87.51) | 1.11 (0.00,288.33) | 1.10 (0.00,284.78) | **Ipra 50mg** |  |  |
| 1.39 (0.31, 6.14) | 0.99 (0.02, 50.07) | 2.73 (0.76, 9.80) | 1.61 (0.47, 5.56) | 1.98 (1.05, 3.74) | 0.81 (0.05, 14.49) | 1.05 (0.33, 3.37) | 0.80 (0.23, 2.78) | 1.07 (0.18, 6.35) | 1.06 (0.18, 6.26) | 1.01 (0.02, 51.02) | 0.99 (0.02, 50.39) | 0.90 (0.02, 46.24) | **Pla** |  |
| 2.76 (0.04,184.71) | 1.97 (0.01,508.89) | 5.44 (0.09,339.22) | 3.21 (0.05,197.96) | 3.96 (0.07,211.93) | 1.62 (0.01,211.52) | 2.09 (0.03,126.06) | 1.60 (0.03, 98.58) | 2.13 (0.03,159.46) | 2.10 (0.03,157.28) | 2.01 (0.01,518.58) | 1.98 (0.01,512.18) | 1.80 (0.01,468.91) | 1.99 (0.04,101.49) | **Tofo 20mg** |

**Footnote:** SGLT-2i: Sodium-glucose co-transporter-2 inhibitors; Cana: Canagliflozin; Dapa: Dapagliflozin; Ertu: Ertugliflozin; Ipra: Ipragliflozin; Hena: Henagliflozin; Bexa: Bexagliflozin; Empa: Empagliflozin; Tofo: Tofogliflozin; Pla: Placebo; DKA: diabetic ketoacidosis.

### **12.4 Exclusion of the risk of bias arising from the randomization process was ‘Some concerns’**

Outcome: The risk of DKA (odds ratio; 95% confidence interval)

| **Active** |  |  |  |  |  |  |  |  |  |  |  |  |  |  |
| --- | --- | --- | --- | --- | --- | --- | --- | --- | --- | --- | --- | --- | --- | --- |
| 1.14 (0.02, 69.73) | **Bexa 20mg** |  |  |  |  |  |  |  |  |  |  |  |  |  |
| 0.49 (0.10, 2.29) | 0.43 (0.01, 25.92) | **Cana 100mg** |  |  |  |  |  |  |  |  |  |  |  |  |
| 0.60 (0.14, 2.64) | 0.53 (0.01, 31.64) | 1.24 (0.32, 4.86) | **Cana 300mg** |  |  |  |  |  |  |  |  |  |  |  |
| 1.61 (0.29, 8.82) | 1.41 (0.02, 97.38) | 3.32 (0.47, 23.25) | 2.67 (0.40, 17.91) | **Dapa 10mg** |  |  |  |  |  |  |  |  |  |  |
| 2.30 (0.04,141.30) | 2.02 (0.01,521.85) | 4.74 (0.08,290.82) | 3.82 (0.06,230.36) | 1.43 (0.02, 99.27) | **Dapa 5mg** |  |  |  |  |  |  |  |  |  |
| 0.84 (0.18, 3.90) | 0.73 (0.01, 45.51) | 1.73 (0.31, 9.64) | 1.39 (0.26, 7.40) | 0.52 (0.07, 3.71) | 0.36 (0.01, 22.70) | **Empa 10mg** |  |  |  |  |  |  |  |  |
| 1.10 (0.24, 4.99) | 0.96 (0.02, 60.75) | 2.27 (0.39, 13.02) | 1.83 (0.33, 9.99) | 0.68 (0.09, 4.95) | 0.48 (0.01, 30.31) | 1.31 (0.33, 5.26) | **Empa 25mg** |  |  |  |  |  |  |  |
| 0.80 (0.15, 4.46) | 0.71 (0.01, 48.87) | 1.66 (0.24, 11.72) | 1.34 (0.20, 9.03) | 0.50 (0.06, 4.32) | 0.35 (0.01, 24.38) | 0.96 (0.13, 6.89) | 0.73 (0.10, 5.33) | **Ertu 15mg** |  |  |  |  |  |  |
| 1.10 (0.18, 6.63) | 0.96 (0.01, 68.13) | 2.26 (0.30, 16.84) | 1.82 (0.26, 13.00) | 0.68 (0.07, 6.22) | 0.48 (0.01, 33.99) | 1.31 (0.17, 9.92) | 1.00 (0.13, 7.69) | 1.36 (0.30, 6.30) | **Ertu 5mg** |  |  |  |  |  |
| 1.12 (0.02, 68.40) | 0.98 (0.00,252.99) | 2.31 (0.04,140.78) | 1.86 (0.03,111.51) | 0.70 (0.01, 48.06) | 0.49 (0.00,126.04) | 1.34 (0.02, 82.74) | 1.02 (0.02, 64.17) | 1.39 (0.02, 96.39) | 1.02 (0.01, 72.22) | **Hena 10mg** |  |  |  |  |
| 1.13 (0.02, 69.25) | 0.99 (0.00,256.14) | 2.34 (0.04,142.53) | 1.88 (0.03,112.89) | 0.70 (0.01, 48.66) | 0.49 (0.00,127.61) | 1.35 (0.02, 83.77) | 1.03 (0.02, 64.97) | 1.41 (0.02, 97.59) | 1.03 (0.01, 73.11) | 1.01 (0.02, 51.34) | **Hena 5mg** |  |  |  |
| 0.94 (0.02, 48.88) | 0.83 (0.00,247.02) | 1.94 (0.03,135.24) | 1.56 (0.02,106.05) | 0.59 (0.01, 43.15) | 0.41 (0.00,123.05) | 1.12 (0.02, 77.82) | 0.86 (0.01, 58.79) | 1.17 (0.02, 86.60) | 0.86 (0.01, 65.74) | 0.84 (0.00,251.72) | 0.83 (0.00,248.62) | **Ipra 50mg** |  |  |
| 1.13 (0.33, 3.83) | 0.99 (0.02, 50.07) | 2.32 (0.69, 7.85) | 1.87 (0.59, 5.97) | 0.70 (0.14, 3.43) | 0.49 (0.01, 24.99) | 1.34 (0.38, 4.78) | 1.02 (0.27, 3.85) | 1.40 (0.28, 6.91) | 1.03 (0.20, 5.36) | 1.01 (0.02, 51.02) | 0.99 (0.02, 50.39) | 1.20 (0.02, 74.69) | **Pla** |  |
| 2.25 (0.04,137.74) | 1.97 (0.01,508.89) | 4.63 (0.08,283.48) | 3.73 (0.06,224.54) | 1.40 (0.02, 96.77) | 0.98 (0.00,253.54) | 2.68 (0.04,166.61) | 2.04 (0.03,129.22) | 2.79 (0.04,194.07) | 2.05 (0.03,145.40) | 2.01 (0.01,518.58) | 1.98 (0.01,512.18) | 2.38 (0.01,715.59) | 1.99 (0.04,101.49) | **Tofo 20mg** |

**Footnote:** SGLT-2i: Sodium-glucose co-transporter-2 inhibitors; Cana: Canagliflozin; Dapa: Dapagliflozin; Ertu: Ertugliflozin; Ipra: Ipragliflozin; Hena: Henagliflozin; Bexa: Bexagliflozin; Empa: Empagliflozin; Tofo: Tofogliflozin; Pla: Placebo; DKA: diabetic ketoacidosis.

### **12.5 Exclusion of studies that the risk of DKA was 0 percent**

Outcome: The risk of DKA (odds ratio; 95% confidence interval)

| **Active** |  |  |  |  |  |  |  |  |
| --- | --- | --- | --- | --- | --- | --- | --- | --- |
| 0.68 (0.12, 3.83) | **Cana 100mg** |  |  |  |  |  |  |  |
| 0.89 (0.18, 4.52) | 1.31 (0.33, 5.26) | **Cana 300mg** |  |  |  |  |  |  |
| 0.99 (0.20, 4.80) | 1.45 (0.35, 6.08) | 1.11 (0.29, 4.20) | **Dapa 10mg** |  |  |  |  |  |
| 1.53 (0.22, 10.37) | 2.24 (0.33, 15.13) | 1.71 (0.27, 10.71) | 1.54 (0.32, 7.49) | **Empa 10mg** |  |  |  |  |
| 2.51 (0.36, 17.59) | 3.68 (0.52, 25.86) | 2.81 (0.43, 18.34) | 2.53 (0.49, 12.94) | 1.64 (0.31, 8.72) | **Empa 25mg** |  |  |  |
| 0.33 (0.01, 8.24) | 0.49 (0.01, 18.72) | 0.38 (0.01, 13.63) | 0.34 (0.01, 12.00) | 0.22 (0.01, 9.16) | 0.13 (0.00, 5.67) | **Ertu 15mg** |  |  |
| 1.03 (0.02, 51.78) | 1.50 (0.02,109.35) | 1.15 (0.02, 80.23) | 1.03 (0.02, 70.88) | 0.67 (0.01, 52.78) | 0.41 (0.01, 32.61) | 3.06 (0.12, 75.35) | **Ertu 5mg** |  |
| 1.93 (0.43, 8.67) | 2.84 (0.78, 10.34) | 2.17 (0.66, 7.07) | 1.95 (1.03, 3.70) | 1.26 (0.30, 5.42) | 0.77 (0.17, 3.49) | 5.77 (0.17,198.46) | 1.88 (0.03,125.67) | **Pla** |

**Footnote:** SGLT-2i: Sodium-glucose co-transporter-2 inhibitors; Cana: Canagliflozin; Dapa: Dapagliflozin; Ertu: Ertugliflozin; Ipra: Ipragliflozin; Hena: Henagliflozin; Bexa: Bexagliflozin; Empa: Empagliflozin; Tofo: Tofogliflozin; Pla: Placebo; DKA: diabetic ketoacidosis.

## Supplementary Figure 1. Inconsistency (incoherence) assessments for different kinds of active antidiabetic drugs


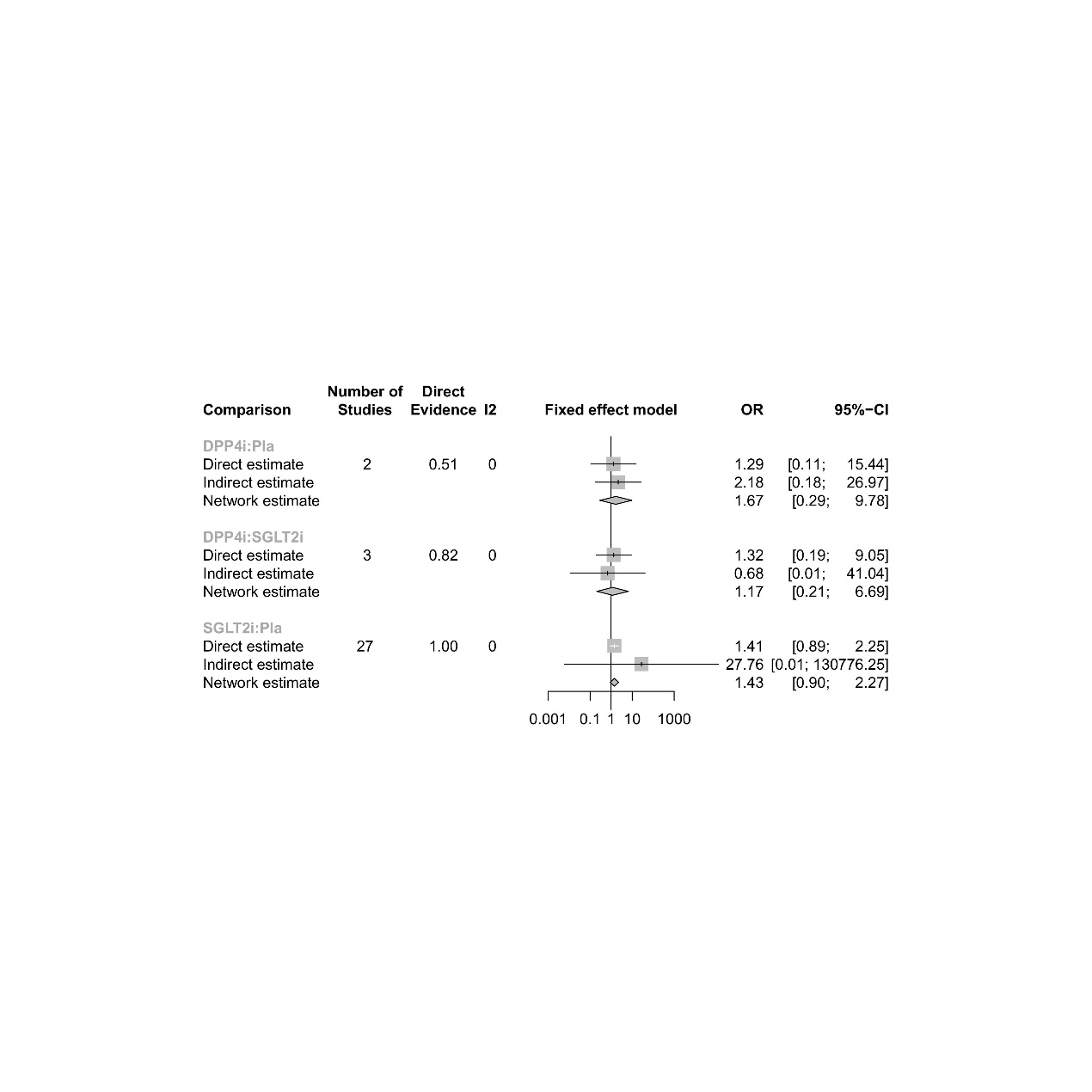


## Supplementary Figure 2. Inconsistency (incoherence) assessments for different doses of SGLT-2i


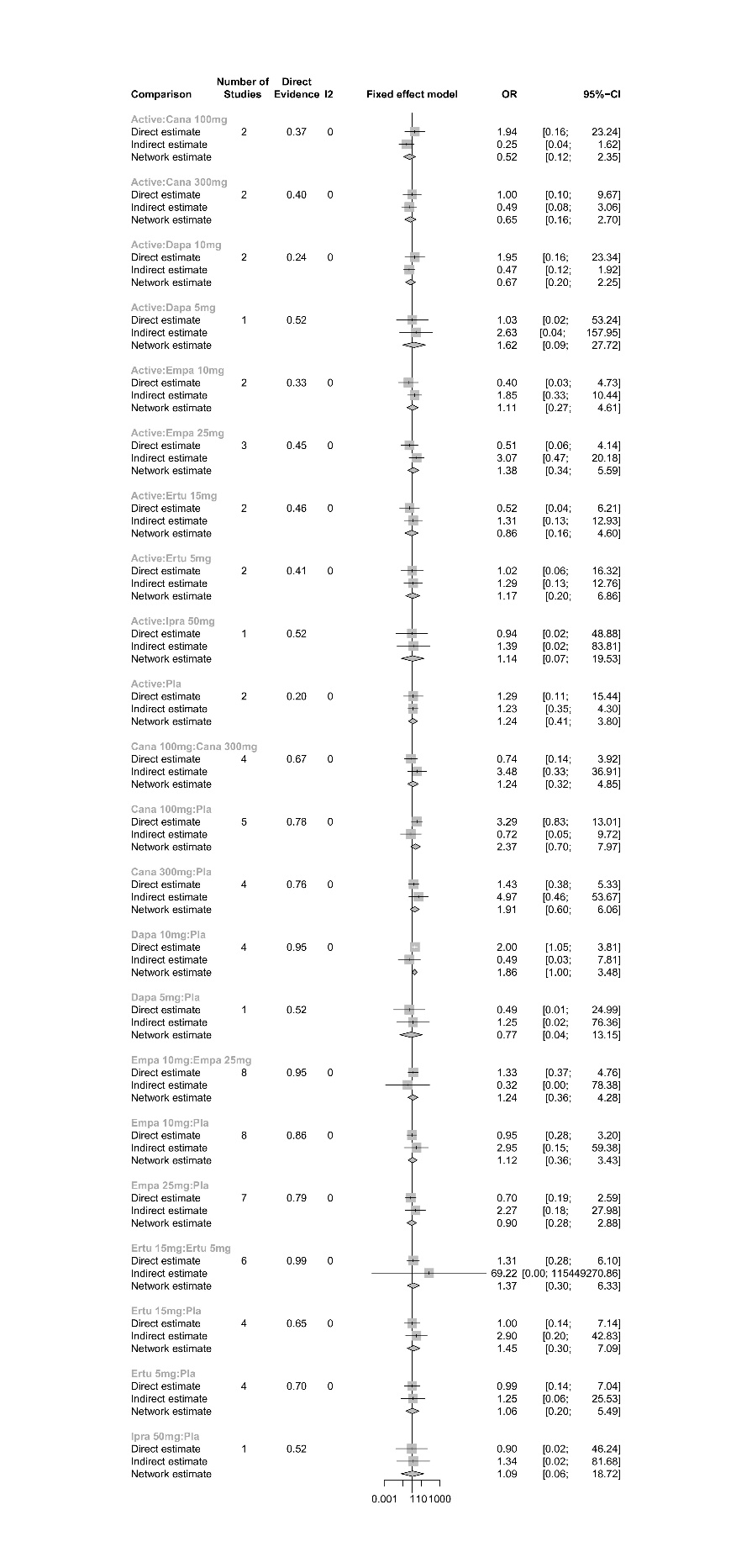


## Supplementary Figure 3. Intransitivity assessments-age at baseline


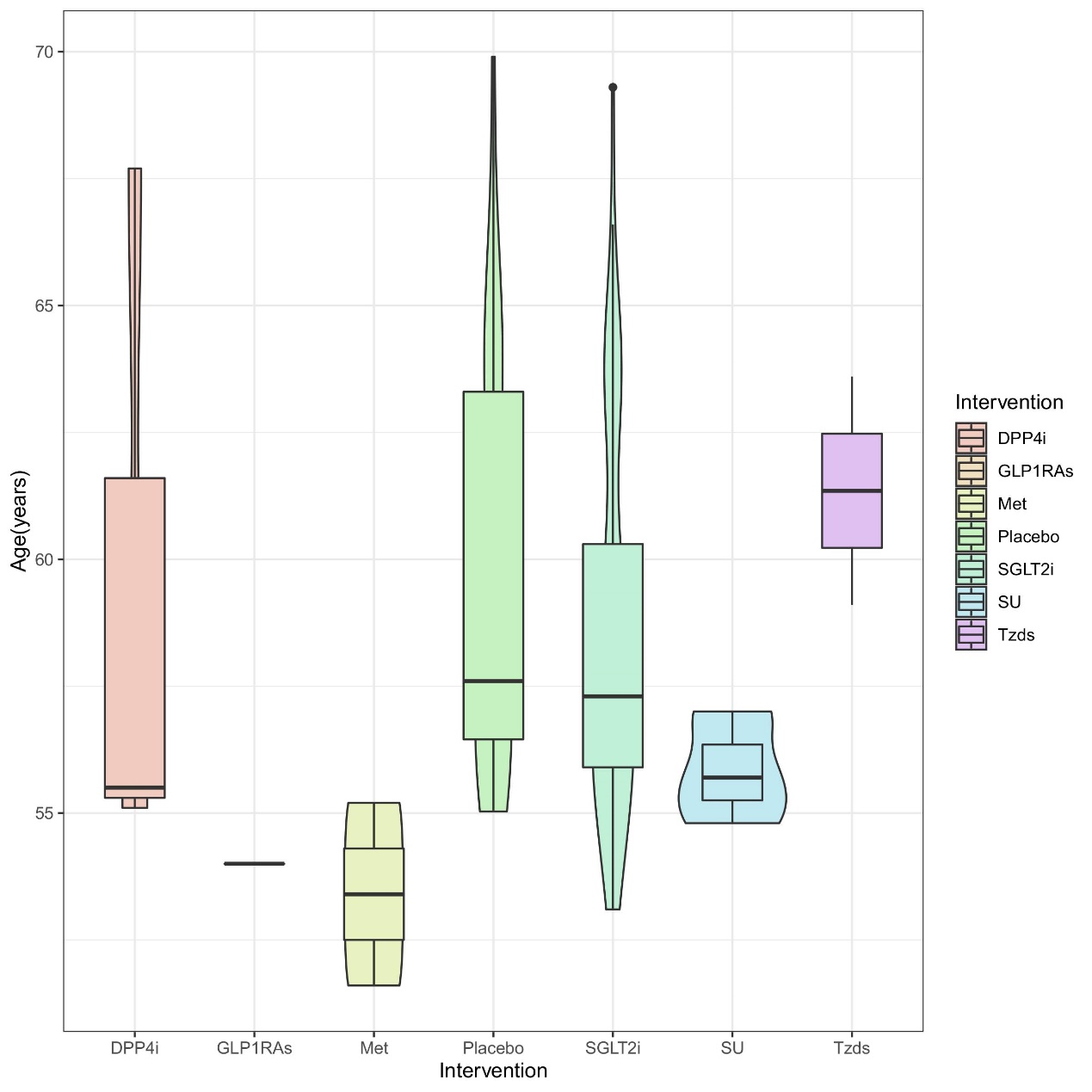


## Supplementary Figure 4. Intransitivity assessments-BMI at baseline


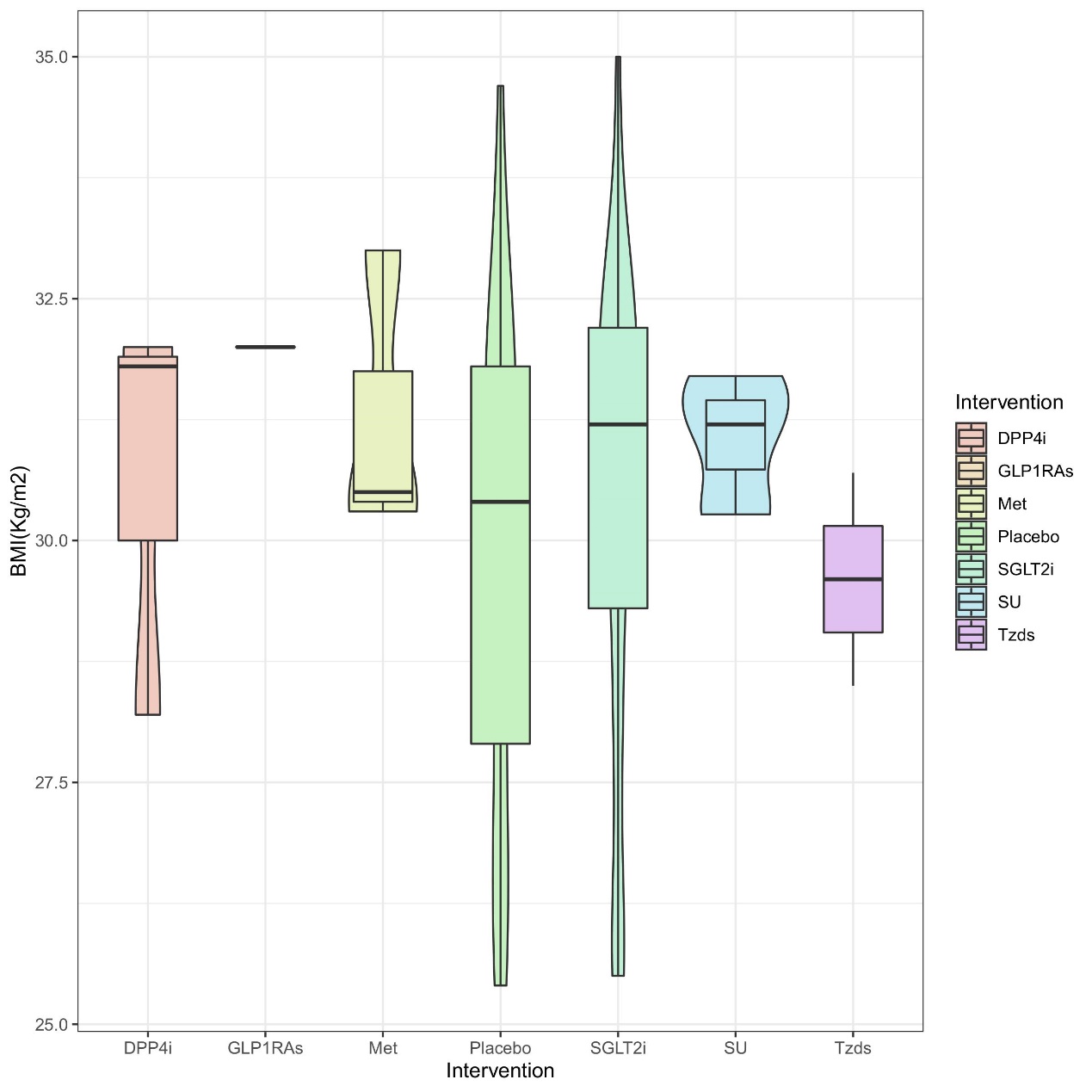


## Supplementary Figure 5. Intransitivity assessments-HbA1c at baseline


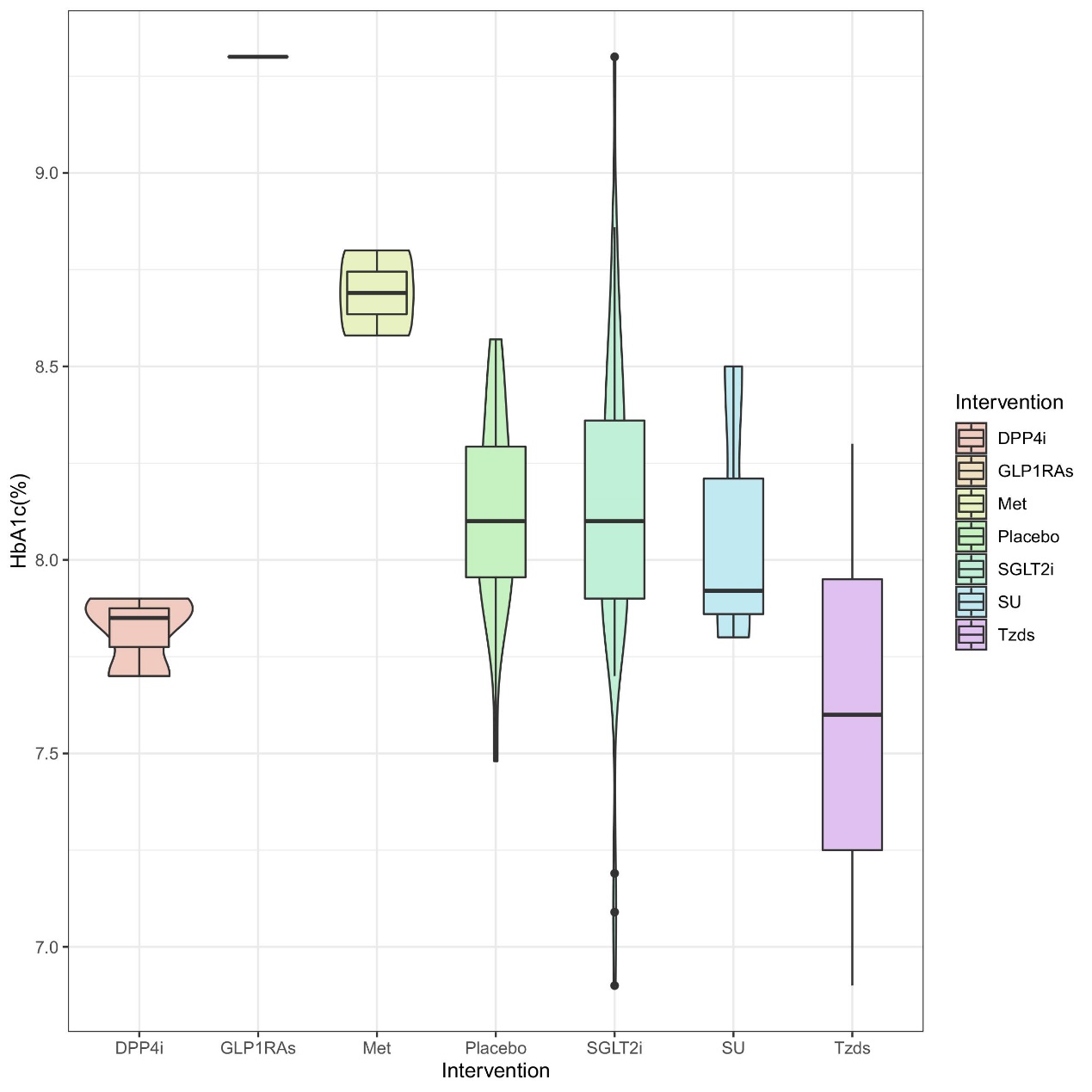


## Supplementary Figure 6. Funnel plot for different doses of SGLT-2i


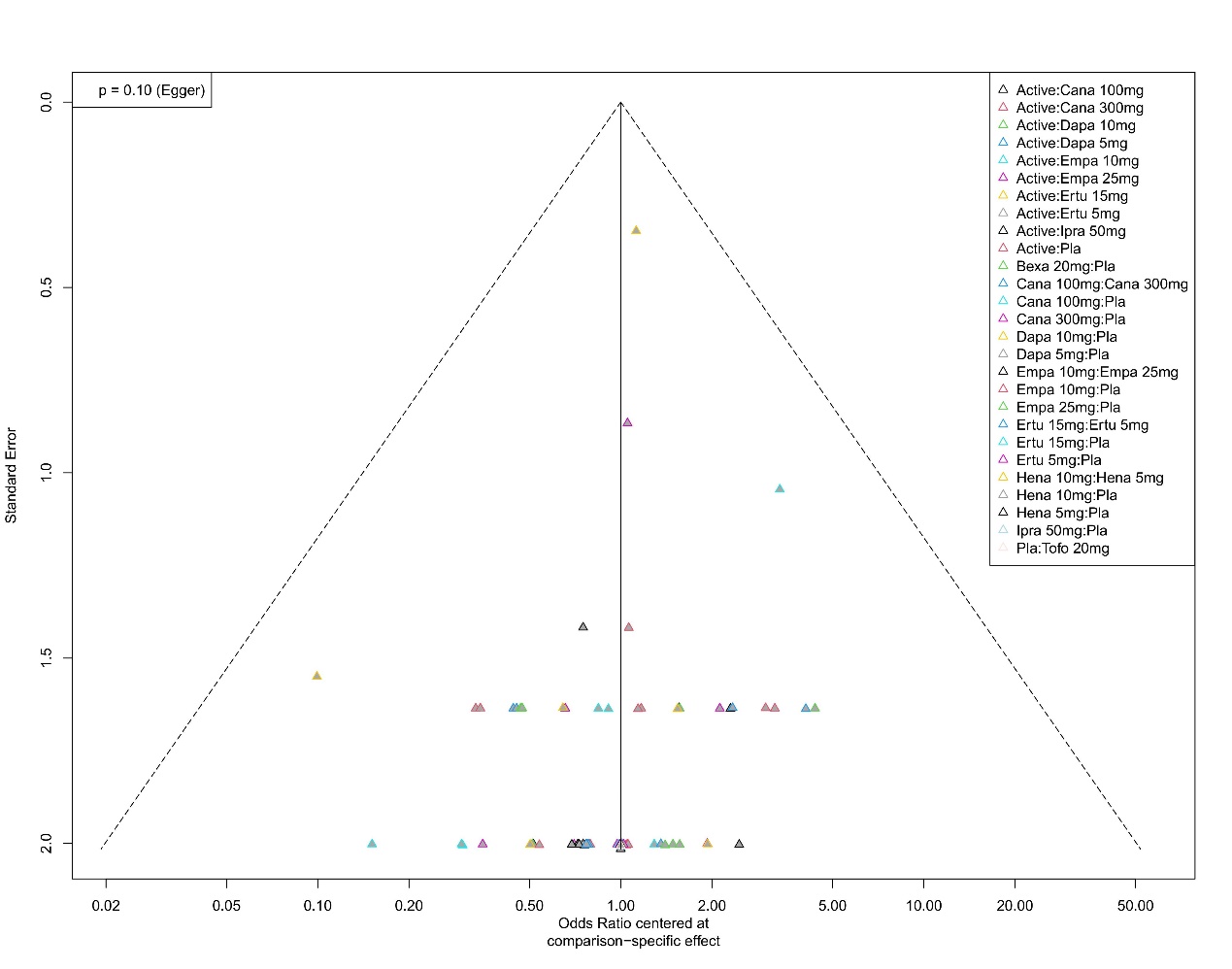


## Supplementary Figure 7. Funnel plot for different kinds of active antidiabetic drugs


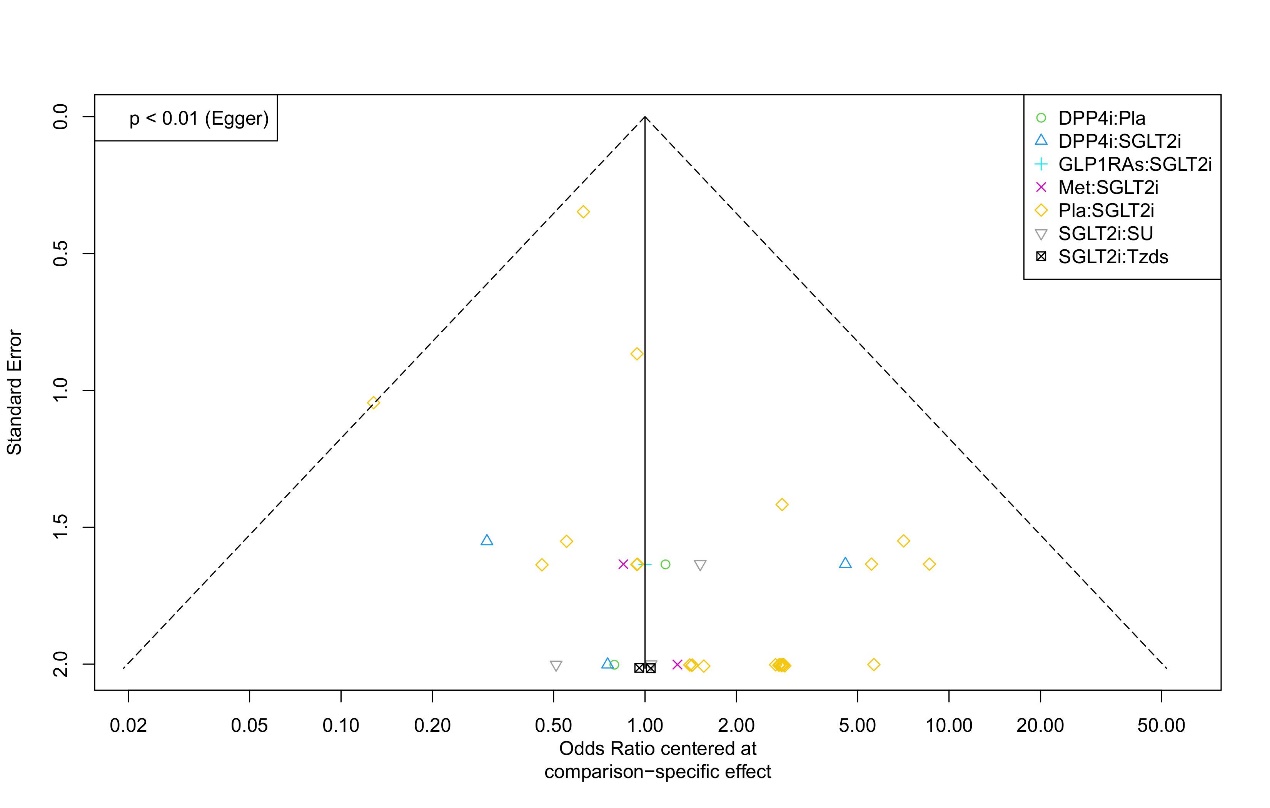

Supplement: Supplementary file 1 [file Table1.docx]
